# Heritable immunization of mice against Lyme disease enables ecological disease prevention

**DOI:** 10.1038/s41467-026-71757-6

**Published:** 2026-04-28

**Authors:** Joanna Buchthal, Emma J. Chory, Zachary Hill, Yu Zhou, Devanand Bondage, Summer DeAmelio, Julien Freeman, Rudolf Jaenisch, Styliani Markoulaki, Wayne A. Marasco, Sam R. Telford, Kevin M. Esvelt

**Affiliations:** 1 Media Laboratory, Massachusetts Institute of Technology, Cambridge, MA USA; 2 Mice Against Ticks, Lincoln, MA USA; 3 Department of Biomedical Engineering, Duke University, Durham, NC USA; 4 Department of Cancer Immunology & Virology, Dana-Farber Cancer Institute, Harvard Medical School, Boston, MA USA; 5 Department of Medicine, Harvard Medical School, Boston, MA USA; 6 Whitehead Institute for Biomedical Research, Cambridge, MA USA; 7 Department of Biology, Massachusetts Institute of Technology, Cambridge, MA USA; 8 Department of Infectious Disease and Global Health, Tufts University, Grafton, MA USA

**Keywords:** Environmental biotechnology, Synthetic biology, Applied immunology, Bacterial infection, Ecological epidemiology

## Abstract

Heritable immunization is a promising approach to controlling infectious diseases by embedding immunity directly into the genomes of wild species that spread human pathogens. Here, we report the genetic engineering of *Mus musculus* to genomically encode a single-chain antibody against *Borrelia burgdorferi*, the causative agent of Lyme disease. After optimization of the antibody format, engineered mice stably produce a LA-2 scFv-albumin fusion protein targeting the *Borrelia* outer surface protein A (OspA) across multiple generations, demonstrating robust heritability and stability of gene expression. Following sequential challenges with infected and uninfected ticks, heterozygous mice exhibit strong resistance to infection, effectively interrupting the *Borrelia burgdorferi* disease transmission cycle. Having recently established protocols to genetically engineer the white-footed mouse *Peromyscus leucopus*, a key reservoir of Lyme disease, these findings demonstrate the feasibility of heritable immunization as a potential strategy for mitigating Lyme disease transmission in the environment. Engineered reservoir immunity may offer a promising approach to controlling vector-borne and zoonotic disease.

## Abstract (web summary)

The wildlife reservoir Peromyscus leucopus – the white-footed mouse - is a primary driver of Lyme disease. Here, authors engineer mice to express a neutralizing anti-OspA antibody, blocking Borrelia infection in vivo and demonstrating heritable immunity as a viable intervention to break the tick-mouse transmission cycle.

## Introduction

Heritable immunization—the encoding of pathogen resistance into the genome of organisms—offers an alternative strategy for controlling infectious diseases by integrating immune protection directly into the germline of species that transmit pathogens. This strategy enables the stable transmission of immunity across multiple generations by providing continuous, systemic protection against pathogens in reservoir hosts. For example, encoding neutralizing antibodies within the genome could disrupt disease transmission cycles at their source, particularly in well-characterized host–pathogen systems. Unlike conventional vaccination, which requires repeated administration to each generation, engineering reservoir species could synergize with concomitant interventions by providing a perpetuating mode of reducing enzootic transmission of infections, particularly those maintained by rodents.

Lyme disease, the most common vector-borne disease in the United States, is a multisystem infection with a global public health impact. The transmission cycle involves a complex interplay between tick vectors, reservoir hosts, and the environment^1^. Infection is not vertically transmitted; rather, infections are transmitted between each new generation of reservoirs and tick vectors. On the East Coast of the United States, *Peromyscus* species, particularly the white-footed mouse (*Peromyscus leucopus*), serve as the primary reservoir for *Borrelia burgdorferi*^2^. In Europe, the bank vole (*Myodes glareolus*) and the wood mouse (*Apodemus sylvaticus*) are key reservoirs for *Borrelia afzelii*^3^, while in Asia, the striped field mouse (*Apodemus agrarius*) serves as a reservoir of *Borrelia garinii*^4^. Additionally, *Mus* species, including *Mus musculus*, have been identified as potential reservoirs of *Borrelia* in Europe^5^.

Given the key role played by rodents in the transmission of Lyme disease, many prevention strategies target these hosts to disrupt the transmission cycle. For example, tick control tubes have been deployed to reduce tick populations and interrupt transmission by distributing permethrin-treated cotton, which mice incorporate into their nests^6,7^. Several additional strategies targeting rodent reservoirs focus on *Borrelia*’s major outer surface protein A (OspA), a key protective antigen expressed by *B. burgdorferi sensu lato* (s.l.). Immunization with OspA induces an antibody response that provides protection by a unique mode of action: anti-OspA antibody ingested by an infecting tick is thought to incapacitate or destroy bacterial spirochetes in the tick prior to their attaining infectivity, thereby preventing transmission^8^. Parenteral immunization of wild white-footed mice with an OspA subunit vaccine in field studies demonstrated a reduction in the prevalence of infected ticks^9^. Oral vaccination with recombinant OspA induces a protective response in mouse models^10^, and a commercially available baited rOspA vaccine has been field tested and may be effective in reducing *B. burgdorferi* transmission in wild mice^11^.

Building on the success of previous OspA-based immunization strategies, we explore the use of genetic engineering to alter the reservoir capacity of key hosts by encoding OspA-targeting antibodies in the mouse genome. This approach, inspired by vector-borne disease control strategies such as the genetic engineering of mosquitoes to combat malaria^12,13^, introduces the concept of heritable immunization—embedding antibody-based immunity into the germline to achieve lasting resistance. While mice have been engineered to produce human antibodies, such as the XenoMouse® and HuMAb Mouse®, these models are used for therapeutic monoclonal antibody discovery and production. They do not produce preexisting, pathogen-specific antibodies, nor are they designed to confer lifelong resistance to specific infections or transmit immunity to offspring^14,15^. Unlike prior research where engineered mouse mothers transferred disease resistance to their pups through antibodies in breast milk^16^, our approach aims to establish continuous systemic protection in the reservoir species, persisting throughout their lifespan, and lasting for many generations in the environment.

In this study, transgenic *Mus musculus* are engineered to express LA-2, a *Mus*-derived monoclonal antibody targeting OspA^17^, with the aim of disrupting Lyme disease transmission. LA-2 is chosen because it has been extensively characterized at both the structural^18^ and functional levels^19^, and confers protection when present during tick feeding, a feature well aligned with heritable immunization^8^. Initial in vitro experiments are conducted to optimize genetic engineering efficiency, antibody design, and stabilization via leader sequences and bicistronic elements. Our original goal was to achieve liver-specific expression, but the initial full-length LA-2-expressing models exhibited insufficient immunity. This led to the development of a mouse model in which the LA-2 antibody is reformatted into a single-chain variable fragment (scFv), fused to albumin for enhanced stability and constitutive, ubiquitous expression from the *Rosa26* locus. These engineered mice exhibit robust antibody production across multiple generations, leading to a statistically significant reduction in the transmission of disease-causing bacteria following exposure to infected ticks. These results establish heritable immunization as a viable strategy for the prevention of diseases with mammalian reservoir species.

## Results

### Testing albumin expression machinery

To develop a heritable immunization strategy against Lyme disease, we began by optimizing the expression of the anti-*Borrelia burgdorferi* antibody LA-2 in mouse hepatocytes. We aimed to minimize unintended physiological effects by restricting antibody expression to a specific tissue while leveraging the native albumin expression machinery to drive antibody secretion into the bloodstream. Specifically, we investigated whether the addition of the minimal albumin promoter was sufficient to drive protein expression when inserted proximal and divergent to the endogenous bi-directional albumin enhancer, or if an additional albumin enhancer was also needed^20^.

We generated two distinct CRISPR-modified cell lines: (1) the first expressing tdTomato from the minimal promoter alone and (2) a second which incorporated both the albumin enhancer and minimal promoter (Supplementary Fig. 1A). In both cell lines, the tdTomato expression cassette was integrated ~300 bp proximal to the native albumin enhancer. RT-qPCR analysis revealed comparable levels of tdTomato and native albumin mRNA expression in the first cell line without the added enhancer (Supplementary Fig. 1B), suggesting that this more minimal design was capable of driving robust expression at levels comparable to endogenous albumin. Given the already high abundance of albumin in circulation, inclusion of the additional enhancer raised concerns about potential antibody overexpression. We therefore selected the first design for in vivo studies.

### Leader sequence selection

We next aimed to identify a leader sequence that could efficiently direct the anti-*Borrelia burgdorferi* LA-2 antibody for secretion into the bloodstream. To optimize LA-2 secretion, we evaluated potential leader sequences using both in silico and in vitro approaches. Using IMGT’s V-Quest tool, we identified candidate leader sequences based on the homology of their associated variable gene segments to the LA-2 heavy chain variable domain, and additionally analyzed leader sequences from albumin, alpha-fetoprotein, and fibronectin (Supplementary Fig. 1C). Cleavage efficiency was assessed using SignalP software to predict the likelihood of proper cleavage between a given leader sequence and the LA-2 antibody sequence (Supplementary Fig. 1C). From these analyses, two leader sequences were selected for further testing. To assess antibody production from the selected leaders, we designed three expression vectors each containing a reformatted version of LA-2 as an scFv-Fc fusion protein, paired with either one of the two selected leader sequences, or no leader sequence (Supplementary Fig. 1D). These constructs were transiently expressed in cultured cells, and antibody secretion was quantified by ELISA over 48 hours. While both leader sequences resulted in expression, one yielded slightly higher expression and was therefore selected for all transgenic mouse designs (chosen leader: MAWVWTLLFLMAAAQIQA) (Supplementary Fig. 1E).

### Bicistronic element selection

To achieve expression of the full-length LA-2 antibody, we utilized bicistronic elements commonly employed in antibody expression cassettes to produce both heavy and light chains without additional expression machinery. We evaluated several 2A peptides and internal ribosome entry sites (IRES) to determine which element best facilitated the coordinated expression of both chains in vitro. We built five expression constructs that each featured a cytomegalovirus (CMV) promoter driving expression of the LA-2 heavy chain adjacent to a distinct 2 A or an IRES sequence, followed by the LA-2 light chain. Constructs were transiently transfected, and ELISAs were performed on antibody supernatant to evaluate antibody concentration and binding. T2A was chosen from the elements tested based on its overall performance and robustness rather than maximal binding alone (Supplementary Fig. 1F).

### Design validation and guide testing

To finalize our mouse design and identify the optimal guide for targeting the albumin locus, we generated four CRISPR-modified cell lines incorporating key design elements from previous constructs along with the full-length LA-2 antibody (Supplementary Fig. 1G). We performed RT-qPCR to measure LA-2 mRNA levels, along with native albumin, our selection marker (puromycin), and the housekeeping gene *β-actin* (Supplementary Fig. 1H), and evaluated antibody production by performing ELISAs on the supernatant from the four LA-2 CRISPR cell lines (Supplementary Fig. 1I).

### Generation and validation of liver-specific full-length LA-2 expressing mice

**Fig. 1:**
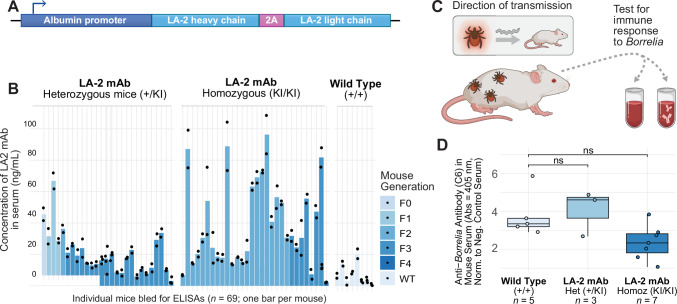
Generation, validation, and infection challenge of liver-specific full-length LA-2-expressing mice. **A** Schematic of the construct introduced into the mouse genome, 300 bp proximal to the native albumin enhancer. The minimal albumin promoter drives the expression of the full-length LA-2 monoclonal antibody. **B** Quantification of LA-2 antibody concentration in the serum of heterozygous (+/KI), homozygous (KI/KI), and wild-type (+/+) mice across multiple generations (F0 through F4). **C** Diagram of the tick challenge. Uninfected engineered and control mice were exposed to *Borrelia burgdorferi*-infected ticks, and post-infestation serum was evaluated for anti-*Borrelia* antibodies to determine infection status. **D** Endpoint ELISA results from transgenic and wild-type mice 21 days post-infestation, detecting *B. burgdorferi*-specific antibodies against C6, a sensitive marker of infection. Statistical significance was determined using unadjusted, two-sided Welch’s t-tests. Box plot statistics: wild type (+/+): minima: 2.908, maxima: 5.870, median: 3.345, bounds of box: 3.195 to 3.640, bounds of whiskers: 2.908 to 3.640; heterozygous (+/KI): minima: 2.680, maxima: 4.879, median: 4.608, bounds of box: 3.644 to 4.744, bounds of whiskers: 2.680 to 4.879; homozygous (KI/KI): minima: 1.065, maxima: 3.843, median: 2.334, bounds of box: 1.809 to 2.817, bounds of whiskers: 1.065 to 3.843. Abs absorbance, Norm. normalized, Neg. negative. Individual biological replicates are shown as circles. Created in BioRender. Buchthal, J. (2026) https://BioRender.com/4mvkhdp. Source data are provided as a Source Data file.

To generate mice expressing the full-length LA-2 antibody, we next constructed a vector containing the aforementioned components, but without a selection marker (Fig. 1A). We performed pronuclear injection to introduce SpCas9, guide 3, and the full-length LA-2 expression cassette into BDF1 mouse embryos. Of the 45 pups delivered, sequencing confirmed that two carried a complete LA-2 antibody knock-in, with one founder exhibiting the correct insertion at the target locus.

Engineered mice were bred for multiple generations to assess both the heritability and stability of LA-2 antibody expression in homozygous and heterozygous mice. OspA ELISAs were performed to quantify the concentration of LA-2 antibodies that correctly bound the protective epitope on OspA (Fig. 1B). However, antibody levels differed widely between homozygous mice, ranging from negligible, wild-type-equivalent levels to over 80 ng/mL.

### Testing the susceptibility of liver-specific full-length LA-2 expressing mice to infection

To evaluate the efficacy of liver-specific, full-length LA-2 antibodies in conferring heritable resistance to *Borrelia burgdorferi* infection, we challenged engineered mice from different filial generations and genotypes with *Borrelia*-infected *Ixodes dammini* nymphs (Fig. 1C). Infected nymphs were prepared by feeding larval *Ixodes dammini* on mice infected with the low-passage N40 *Borrelia burgdorferi* strain (wild type)^8^. All infected nymphs were derived from a single vial with a 70% infection rate. During infestation, 10 nymphs were applied to the ears and nape of the neck of each anesthetized mouse. After 21 days, serum was collected and analyzed using an endpoint ELISA to detect *B. burgdorferi*-specific antibodies against C6, a sensitive marker of infection^21^. Although some homozygous mice exhibited very low or undetectable C6 antibody levels, suggesting potential immunity, neither the homozygous nor heterozygous mice showed a statistically significant reduction in anti-C6 antibody levels compared to wild-type controls (Fig. 1D). Together, these intermediate results revealed key design constraints, established the framework used to evaluate immunity, and guided the development of the architecture described below.

### Generation of *Rosa26*-targeted LA-2 scFv-albumin mice

To develop a mouse model with enhanced antibody expression and improved resistance to infection, we reformatted the full-length LA-2 antibody into a single-chain variable fragment (scFv) to address the expression challenges associated with bicistronic elements and to prevent the mispairing of the full-length LA-2 heavy and light chains with endogenous antibodies. During reformatting, we tested binding and observed an almost one-log reduction in affinity when converting the full-length LA-2 IgG to an scFv with a Vh-(G4S)3-Vl linker (Supplementary Fig. 2).

To optimize LA-2 scFv expression, stability, and binding, we evaluated 15 different linker sequences by generating expression constructs each with a unique linker positioned between the LA-2 scFv-Fc heavy and light chains (Supplementary Fig. 3A). Three constructs employed multimers of the commonly used GGGGS pentapeptide, while the remaining linkers were designed to modulate flexibility and length by varying the number of glycine and serine residues. The top-performing linker, GGGGSGGGGSGGGGSGGGG, demonstrated superior binding characteristics to purified full-length LA-2 and was selected for mouse model creation (Supplementary Fig. 3B).

**Fig. 2:**
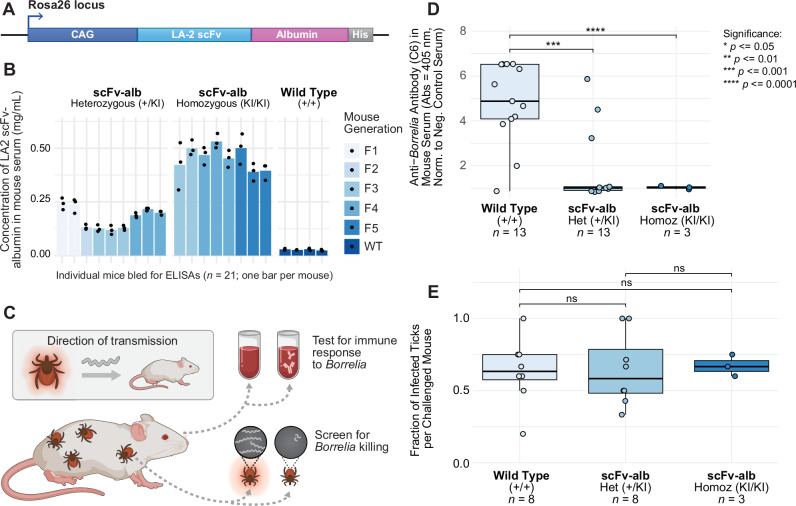
Generation, validation, and infection challenge of LA-2 scFv-albumin expressing mice. **A** Schematic representation of the transgene inserted into the *Rosa26* locus, encoding the LA-2 scFv fused to mouse albumin under the control of the CAG promoter. **B** Serum concentrations of LA-2 scFv-albumin in heterozygous (+/KI), homozygous (KI/KI) and wild type (+/+) mice across multiple generations, measured by ELISA. **C** Illustration of the tick challenge: Uninfected engineered and control mice were exposed to *Borrelia burgdorferi*-infected ticks. Post-infestation, serum was analyzed for signs of infection, and ticks were evaluated for infection status. **D** Endpoint ELISA results from transgenic and wild-type mice 21 days post-infestation, detecting *B. burgdorferi*-specific antibodies against C6, a sensitive marker of infection. Asterisks denote statistical significance compared to wild type (+/+) mice, determined using unadjusted, two-sided Welch’s t-tests (***p = 0.000179; ****p = 0.000007). Box plot statistics: wild type (+/+): minima: 0.868, maxima: 6.539, median: 4.880, bounds of box: 4.089 to 6.523, bounds of whiskers: 0.868 to 6.539; heterozygous (+/KI): minima: 0.835, maxima: 5.869, median: 1.008, bounds of box: 0.901 to 1.065, bounds of whiskers: 0.835 to 1.065; homozygous (KI/KI): minima: 0.950, maxima: 1.099, median: 1.033, bounds of box: 0.992 to 1.066, bounds of whiskers: 0.950 to 1.099. Abs absorbance, Norm. normalized, Neg. negative. **E** Graph showing the fraction of *Borrelia*-infected ticks per individual mouse, comparing ticks that fed on transgenic versus wild-type mice. Engorged ticks were collected, stored, and assessed for the presence or absence of *Borrelia* spirochetes using indirect immunofluorescence. Significance was assessed via two-sided Welch’s t-tests to account for unequal variances, without multiple comparison corrections. Box plot statistics: wild type (+/+): minima: 0.200, maxima: 1.000, median: 0.633, bounds of box: 0.550 to 0.750, bounds of whiskers: 0.500 to 1.000; heterozygous (+/KI): minima: 0.333, maxima: 1.000, median: 0.583, bounds of box: 0.464 to 0.857, bounds of whiskers: 0.333 to 1.000; homozygous (KI/KI): minima: 0.600, maxima: 0.750, median: 0.667, bounds of box: 0.633 to 0.708, bounds of whiskers: 0.600 to 0.750. Individual biological replicates are shown as circles. Created in BioRender. Buchthal, J. (2026) https://BioRender.com/cyyfl61. Source data are provided as a Source Data file.

To generate engineered mice, we next developed a targeting construct with modifications aimed at enhancing antibody expression and stability. Given the inherently short half-life of scFvs, we fused the LA-2 scFv to albumin, a strategy previously explored in therapeutic contexts to improve protein stability and extend half-life^22,23^. To ensure robust antibody production, we utilized the CAG enhancer/promoter, known for driving high gene activity across various loci, and integrated it into the *Rosa26* locus^24^, a well-established safe harbor site known for stable and consistent transgene expression (Fig. 2A).

We performed pronuclear injection to introduce SpCas9, guide RNA, and the LA-2 scFv-albumin expression cassette into embryos from B6 mice. Eighteen pups were produced and tested, one of which exhibited the correct knock-in.

### Testing heritable antibody expression in *Rosa26*-targeted LA-2 scFv-albumin mice

Antibody expression was evaluated by testing serum samples from 6 generations of mice. Engineered mice produced at least 1000-fold more antibody compared to the previous design, with homozygous mice expressing roughly twice the amount seen in heterozygotes (Fig. 2B). Unlike the previous design, there was no variability in antibody production; animals with one copy of the gene consistently produced close to 0.25 mg/mL of antibody, while those with two copies produced approximately 0.5 mg/mL. For context, total IgG concentrations typically range from 10–15 mg/mL in *Mus*; while all transgenes are likely to reduce fitness in the wild by default, these levels suggest that the scFv is unlikely to constitute a major burden. Given the high circulating antibody concentrations observed, size-exclusion chromatography (SEC) was performed on purified LA-2 scFv-albumin to evaluate quaternary structure. The antibody eluted predominantly as a monomer (Supplementary Fig. 4), suggesting that these concentrations are unlikely to drive higher-order oligomerization or aggregation. Importantly, antibody expression was consistent across successive generations, confirming stable, heritable expression.

### Testing the susceptibility of *Rosa26*-targeted LA-2 scFv-albumin mice to infection

To evaluate the resistance of newly engineered LA-2 scFv-albumin-expressing mice to *Borrelia burgdorferi* infection, we challenged these mice and wild-type controls with *B. burgdorferi*-infected nymphs (Fig. 2C). As in the previous challenge, ten nymphs were applied to each mouse and allowed to feed to repletion. Twenty-one days post-challenge, mice were bled, and serum was analyzed for *B. burgdorferi*-specific IgG antibodies against the C6 peptide (Fig. 2D). Both homozygous and heterozygous mice showed a statistically significant reduction in anti-C6 antibody production compared to wild-type controls. Homozygous mice, in particular, demonstrated robust protection from infection. Heterozygous mice also exhibited significant immunity (*p* = 1.8E-4). The small subset exhibiting reduced protection may reflect differences in infected tick or *Borrelia* burdens. Notably, wild mice are seldom captured with more than a few nymphs attached^25^, most of which are not infected, suggesting that this infection challenge is quite stringent relative to what would be expected in the wild^26^. While further studies are required to fully characterize the full extent of protection in both heterozygous and homozygous mice, these results indicate a strong and heritable immune response that guards against infection upon tick challenge, especially in homozygotes.

### Evaluating tick infection following the first challenge with *Rosa26*-targeted LA-2 scFv-albumin mice

Next, we examined the nymphs from the previous challenge for signs of infection to evaluate the borreliacidal activity of the scFv-albumin antibodies in the engineered mice. Engorged nymphs were collected post- infestation and stored for 14 days before analysis^8^. Ticks were homogenized, applied to slides, and stained by indirect immunofluorescence for *B. burgdorferi*. For analysis, each tick was categorized based on the presence or absence of spirochetes, regardless of infection intensity (Fig. 2E). Intriguingly, while the scFv-albumin antibodies protected the mice, they did not appear to reduce the spirochete load in the infected ticks used to challenge those mice, contrary to the previously reported mode of action for anti-OspA antibodies^8^. Further studies to investigate the molecular basis of protection appear warranted.

### Testing *Borrelia* transmission to uninfected ticks feeding on previously challenged *Rosa26*-targeted LA-2 scFv-albumin mice

**Fig. 3:**
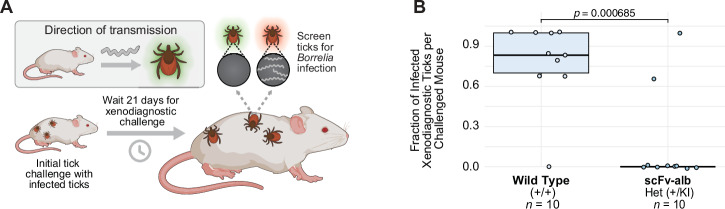
Xenodiagnostic challenge and infection analysis in LA-2 scFv-albumin expressing mice. **A** Schematic of the xenodiagnostic challenge. Uninfected larval ticks were fed on previously challenged *Rosa26*-targeted LA-2 scFv-albumin mice 21 days post-challenge. **B** Fraction of infected ticks per mouse, assessed by indirect immunofluorescence of dissected tick guts post-molt. Detection of *Borrelia burgdorferi* spirochetes in any tick was considered evidence of an infected and infectious mouse. Statistical significance was determined using a two-sided Welch’s t-test (*p* = 0.000685). Box plot statistics: wild type (+/+): minima: 0.000, maxima: 1.000, median: 0.833, bounds of box: 0.667 to 1.000, bounds of whiskers: 0.667 to 1.000; heterozygous (+/KI): minima: 0.000, maxima: 1.000, median: 0.000, bounds of box: 0.000 to 0.000, bounds of whiskers: 0.000 to 0.000. Individual biological replicates are shown as circles. Created in BioRender. Buchthal, J. (2026) https://BioRender.com/bxjqlne. Source data are provided as a Source Data file.

Even without *Borrelia* clearance from already-infected ticks, engineered mice may reduce or block transmission to the next generation of larvae. To assess reservoir competence and the impact of anti-OspA antibodies on *Borrelia* transmission, we performed xenodiagnosis using uninfected larval ticks (Fig. 3A)^27^. Twenty-one days after the challenge with infected ticks, mice were infested with uninfected larval *Ixodes dammini* ticks. Engorged larvae were collected and maintained for 4 to 5 weeks until molting, then dissected and stained via indirect immunofluorescence for *Borrelia burgdorferi*. Detection of spirochetes in any tick (e.g., 1 out of 5) was considered evidence of an infected and infectious mouse. A comparison of ticks feeding on wild-type versus engineered mice revealed a highly significant difference in infection status (*p* = 6.9E−4)(Fig. 3B). 80% of engineered mice previously challenged with a large number of infected ticks were completely free of infection.

## Discussion

By encoding anti-Lyme antibody genes into the germline of *Mus musculus*, we conferred heritable resistance to Lyme disease. After evaluating multiple antibody formats, including full-length and reformatted constructs, the selected antibody design was stably inherited across multiple generations and provided robust immunity to infection. This represents a key in vivo achievement for our longstanding project aiming to advance community-guided ecological engineering^28^. Our results highlight the potential of heritable immunization as a strategy for controlling infectious diseases by genetically engineering reservoir species. These findings lay the groundwork for engineering *Peromyscus leucopus*, the main reservoir in North America, and may have translational relevance in regions where *Mus* is thought to act as a reservoir, such as parts of Europe.

The same approach to engineering heritable immunity could be adapted to target a broad spectrum of vector-borne and zoonotic diseases by introducing specific antibodies or immune-modulating factors into the genomes of key wildlife species. Examples include carriers of hantaviruses^29^, leptospirosis^30^, Lassa fever^31^, and other emerging pathogens. Encoding immune protection directly into the germline could advance global efforts to prevent and control infectious diseases impacting animal and human health.

While our results demonstrate effective immune protection, the precise mechanism remains unclear and warrants further study. We note that tissue spirochete burdens were not assessed by culture or qPCR in this study, as our analyses focused on transmission outcomes rather than host infection burden. Previous work suggested that anti-OspA antibodies directly neutralize *Borrelia* in the tick midgut via direct cellular lysis or complement activation^8^. However, the single-chain antibody we employed did not clear *Borrelia* within the tick, suggesting that an alternative mechanism may be responsible for disrupting the transmission cycle. One plausible explanation, as proposed by Wang et al. (2016), is that anti-OspA antibodies interfere with *Borrelia*’s gene switch from *ospA* to *ospC*, which is essential for spirochete infection of vertebrates^19^. This switch, regulated by environmental signals (temperature, pH, and spirochete density) within the tick during a blood meal, is crucial for the successful infection of a mammalian host^32,33^. By binding to the surface of *Borrelia*, anti-OspA antibodies may prevent the bacteria from reaching the critical density required for the *ospA*/*ospC* gene switch, or alternatively, they may impair the midgut transmigration essential for transmission^33,34^. To this end, more recent work has emphasized that successful transmission requires regulated dissemination of spirochetes from the tick midgut during feeding, and that disruption of this process can substantially impair transmission^34,35^.

Without Fc-mediated effector functions, the LA-2 scFv appears sufficient to prevent *Borrelia* from establishing infection in the mammalian host, highlighting that antibody binding alone, independent of Fc-driven immune mechanisms, could effectively disrupt the transmission cycle. Differences in affinity, avidity, and binding valency between the scFv and the full-length antibody, including reduced agglutinating potential following reformatting, may together contribute to these distinct findings and help explain the absence of detectable borreliacidal activity despite preserved transmission-blocking efficacy. Future iterations incorporating new antibody formats designed to enhance affinity and avidity (as well as the creation of a non*-Borrelia* targeting scFv control mouse for comparison) could be explored for increased efficacy, particularly in applications requiring bactericidal activity or targeting other pathogens, and to further elucidate the underlying mechanisms of protection.

While our findings represent a critical step toward heritable immunization, several challenges remain before implementation. Foremost among these is the need to engineer *Peromyscus leucopus* to carry heritable immunity. We have now established the ability to genetically engineer *Peromyscus*^36^, but antibody constructs have not yet been genomically encoded in this species to recapitulate the effects described here. Extensive field trials and computational modeling are necessary to assess ecological fitness and predict efficacy in natural environments, as fitness costs, though not yet observed in the lab, could manifest in the wild. With the guidance of communities on Nantucket and Martha’s Vineyard, preliminary data collection on multiple candidate uninhabited field trial islands has now been underway for several years. Additionally, while engineering *P. leucopus* may suffice to collapse tick infection levels in environments such as Nantucket where they are the dominant reservoir, the complex ecology of *Borrelia burgdorferi* and the potential role of additional reservoirs elsewhere^37^ suggest that engineering a single species may not fully disrupt transmission in all environments, potentially requiring application of this strategy across multiple hosts. Our recent advances in rodent ovulation tracking, which enable non-invasive identification of fertile windows and facilitate genetic modification in non-model rodents, may open avenues for engineering other non-model species involved in *Borrelia* transmission, offering a potential strategy for controlling Lyme and other diseases involving rodent reservoirs^36^. A release strategy will need to be established as this approach advances beyond laboratory settings. Given the limited dispersal of *Peromyscus leucopus*^38^, population replacement through targeted releases, particularly during seasonal bottlenecks, may be sufficient to achieve high frequencies of protective alleles without the need for gene drive-based approaches. Importantly, protection in heterozygous animals indicates that a single protective allele is sufficient to confer immunity, suggesting that protective effects could be maintained even in the absence of fixation (complete replacement of alternative alleles in the population). Because the intentional release of genetically modified mammals into the environment has no clear regulatory precedent, this application will likely necessitate new regulatory frameworks and adaptive approaches that integrate ecological risk assessment, public health goals, and community governance as this work moves toward real-world implementation.

This study represents a key demonstrative step towards heritable genetic immunization of reservoir species. Future research should focus on refining this approach, elucidating the mechanisms of immune protection, and conducting field trials to evaluate the real-world impact of heritable immunization strategies with the guidance of local communities. The integration of such strategies into broader public health initiatives could prove pivotal in regions where vector-borne diseases threaten human and animal health.

## Methods

### Ethical statement

The Massachusetts Institute of Technology’s Committee on Animal Care (CAC) approved all mouse procedures, and all mice were maintained at MIT (Cambridge, MA, USA) in strict accordance with all institutional protocols, DOD guidelines, and the Guide for the Care and Use of Laboratory Animals. The Tufts University Institutional Animal Care & Use Committee approved all mouse studies performed at Tufts (North Grafton, MA, USA).

### Testing albumin expression machinery

Hepa 1-6 cells (ATCC, Cat# CRL-1830) were co-transfected with two integration cassettes: one containing the minimal albumin promoter driving tdTomato expression and the other incorporating the rat albumin enhancer^39^ in addition to the minimal albumin promoter and tdTomato. Transfections were carried out using Lipofectamine 2000 (Thermo Fisher) and Opti-MEM (Gibco), following standard protocols. SpCas9 and one of four CRISPR guides, targeting sequences approximately 300 bp upstream of the albumin enhancer, were included in the transfection mix. The guide sequences used were as follows:

gRNA 1: GAGCTAACCTTCTGTCCTAG

gRNA 2: GCCTTAGCCAGTGTTTGCAC

gRNA 3: GCCTGTGCAAACACTGGCTA

gRNA 4: GCTGGCTAAGGCATGAACTT

Following transfection, cells were cultured under hygromycin selection until only cells with integrated cassettes remained. After 3 weeks of selection, successful integration was confirmed by PCR. RNA was extracted using TRIzol Reagent (Thermo Fisher), and cDNA synthesis was performed using the Quantitect Reverse Transcription Kit (Qiagen). Expression levels of tdTomato and native albumin mRNA were quantified by RT-qPCR using the SensiFAST SYBR Hi-ROX Kit (Bioline), with mRNA levels normalized to *β-actin*. The following primer sets were used for amplification:

Albumin 1:

Alb-2-qPCR-F: 5’-GACGTGTGTTGCCGATGAGT-3’

Alb-2-qPCR-R: 5’-GTTTTCACGGAGGTTTGGAATG-3’

Albumin 2:

Alb-3-qPCR-F: 5’-TCCAAACCTCCGTGAAAACTATG-3’

Alb-3-qPCR-R: 5’-TGTGTTGCAGGAAACATTCGT-3’

tdTomato 1:

tdTomato-1-qPCR-F: 5’-CTTGTACAGCTCGTCCATGC-3’

tdTomato-1-qPCR-R: 5’-AACTGCCCGGCTACTACTAC-3’

tdTomato 2:

tdTomato-3-qPCR-F: 5’-CGCGCATCTTCACCTTGTAG-3’

tdTomato-3-qPCR-R: 5’-GCGTGATGAACTTCGAGGAC-3’

*β-Actin*:

Bact-1-qPCR-F: 5’-GGCTGTATTCCCCTCCATCG-3’

Bact-1-qPCR-R: 5’-CCAGTTGGTAACAATGCCATGT-3’

### Antibody sequence design

LA-2 heavy- and light-chain variable region sequences were obtained from the published LA-2 antibody structure^18^ and used to generate both full-length and scFv antibody formats.

### Leader sequence selection

Leader sequences for efficient secretion of the anti-*Borrelia burgdorferi* LA-2 antibody were selected through a combination of in silico analysis and in vitro validation. For the in silico analysis, candidate leader sequences were identified using IMGT’s V-Quest tool, and cleavage efficiency was predicted using SignalP software (version 5.0).

For in vitro testing, three expression vectors were constructed: each encoding an identical LA-2 single-chain variable fragment fused to the Fc region (scFv-Fc), paired with either one of the two selected leader sequences or no leader sequence as a control. The following two leader sequences were tested:

IGHV9-2-1*01: MAWVWTLLFLMAAAQIQA

IGHV9-3*02: MDWLWNLLFLMAAAQIQA

Hepa 1-6 cells were transfected with the aforementioned constructs using Lipofectamine 2000 (Thermo Fisher Scientific) according to the manufacturer’s protocol. Supernatants were collected at 0, 24, and 48 hours post-transfection.

LA-2 scFv-Fc secretion was quantified using a mouse IgG2b ELISA kit (Bethyl Laboratories) with a secondary antibody specific to IgG2b for detection. ELISAs were performed on the collected supernatants following the manufacturer’s instructions.

### Bicistronic element selection

To optimize the co-expression of the heavy and light chains of the full-length LA-2 antibody, five expression constructs were designed. Each construct contained a CMV promoter driving the LA-2 heavy chain, followed by either one of four 2A peptide sequences (F2A, E2A, P2A, or T2A) or an internal ribosome entry site (IRES CVEB) to facilitate co-expression of the heavy and light chain.

Hepa 1-6 cells were transfected with the constructs using polyethylenimine (PEI) in Opti-MEM (Gibco) following standard protocols. After 72 hours, the supernatants were collected for analysis. Antibody concentration was quantified using an IgG2a ELISA kit (Bethyl Laboratories, E99-107). For antigen-binding assessments, rOspA (produced by GenScript) was coated onto plates at a concentration of 5 μg/mL. Binding was detected using the IgG2a Bethyl Laboratories ELISA kit (E99-107). Full-length LA-2 IgG2a (produced by GenScript) was used as a standard for quantification at a starting concentration of 500 ng/mL, and as a positive control in the binding assay at a starting concentration of 1 µg/mL.

### Validation and guide testing for full-length LA-2 design

Custom oligonucleotides used in this study were synthesized by commercial vendors: PCR primers for cloning and sequencing were obtained from GenScript, and CRISPR guide RNAs were synthesized by Integrated DNA Technologies (IDT).

Four CRISPR-modified Hepa 1-6 cell lines were generated to validate the full-length LA-2 antibody design and assess the efficiency of different CRISPR guide RNAs. Every cell line contained the full-length LA-2 IgG2a construct along with a puromycin resistance marker to enable selection of LA-2-positive populations.

Hepa 1-6 cells were co-transfected with the LA-2 IgG2a construct and one of four px330 plasmids encoding SpCas9 (Addgene plasmid #42230) and one of the following guide RNAs, targeting a site approximately 300 bp proximal of the albumin enhancer:

gRNA 1: CAGCTAACCTTCTGTCCTAG

gRNA 2: GCCTTAGCCAGTGTTTGCAC

gRNA 3: TCCTGTGCAAACACTGGCTA

gRNA 4: ACTGGCTAAGGCATGAACTT

Transfections were performed using Lipofectamine 2000 (Thermo Fisher) in Opti-MEM (Gibco), following standard protocols. Post-transfection, cells were selected with 2 µg/mL puromycin for three weeks to isolate stably integrated clones.

Total RNA was extracted from the CRISPR-modified Hepa 1–6 cells using the RNeasy Mini Kit (Qiagen), followed by cDNA synthesis with the Quantitect Reverse Transcription Kit (Qiagen). RT-qPCR was performed using the SensiFAST SYBR Hi-ROX Kit (Bioline), with mRNA levels normalized to *β-actin* and baseline expression in unmodified Hepa 1-6 cells. The following primer sets were used for amplification:

LA-2 mAb 1:

LA-2-1-qPCR-F: 5’-CTCCCTGTGGGTCTGAGTTT-3’

LA-2-1-qPCR-R: 5’-CCCATTGTTACATGCGTCGT-3’

LA-2 mAb 2:

LA-2-2-qPCR-F: 5’-TACCTGGTTGCAGGGTTGAT-3’

LA-2-2-qPCR-R: 5’-TCTGGCTTCATGCTCAATGC-3’

Albumin 1:

Alb-1-qPCR-F: 5’-CAAGAGTGAGATCGCCCATCG-3’

Alb-1-qPCR-R: 5’-TTACTTCCTGCACTAATTTGGCA-3’

Albumin 2:

Alb-2-qPCR-F: 5’-TGCTTTTTCCAGGGGTGTGTT-3’

Alb-2-qPCR-R: 5’-TTACTTCCTGCACTAATTTGGCA-3’

Puromycin resistance:

Puro-1-qPCR-F: 5’-CCACACCTTGCCGATGTC-3’

Puro-1-qPCR-R: 5’-CACCGAGCTGCAAGAACTC-3’

*β-Actin*:

Bact-1-qPCR-F: 5’-GGCTGTATTCCCCTCCATCG-3’

Bact-1-qPCR-R: 5’-CCAGTTGGTAACAATGCCATGT-3’

Supernatants were collected 72 hours post-transfection, and antibody levels were measured using the IgG2a ELISA kit (Bethyl Laboratories, E99-107).

### Generation of full-length LA-2 expressing *Mus musculus*

Pronuclear injections were performed by the Whitehead Institute GEM Core. A vector containing the LA-2 expression cassette, without a selectable marker, was microinjected into BDF1 mouse embryos (generated in-house by the Whitehead Institute GEM Core by mating DBA/2N males with C57BL/6N females, with both parental lines originally sourced from Charles River Laboratories) along with SpCas9 and guide RNA 3 (gRNA 3 sequence: TCCTGTGCAAACACTGGCTA), targeting a region 300 bp upstream of the albumin enhancer. Successfully injected embryos were implanted into pseudopregnant females. Of the 45 pups born, two carried the full-length LA-2 knock-in. Sanger sequencing of PCR amplicons confirmed correct integration at the target locus in one of these mice.

### Genotyping full-length LA-2 expressing *Mus musculus*

Genomic DNA was extracted from ear punches of LA-2-expressing *Mus musculus* using the GenElute Mammalian Genomic DNA Mini-prep Kit (Sigma-Aldrich) according to the manufacturer’s protocol. Genotyping was performed via PCR using PrimeSTAR® DNA Polymerase (Takara Bio) with two primer sets: one set amplifying the LA-2 heavy chain [LA-2-Heavy-F (5’-CCCATTGTTACATGCGTCGT-3’) and LA-2-Heavy-R (5’-AGGCATGAAGTCGGTTACCA-3’)], and the other set targeting the wild-type Albumin locus [Alb-F (5’-GCCTCTAATTCCCGTGTTCC-3’) and Alb-R (5’-TTGAACAGCCCACGAGAGAC-3’)]. PCR products were analyzed by agarose gel electrophoresis to assess zygosity. Homozygous mice, with complete integration of the LA-2 construct at both alleles, exhibited no amplification with the Albumin primers due to the size of the PCR product. Heterozygous mice displayed amplification from both sets of primers.

### Mus musculus husbandry

*Mus musculus* (substrains C57BL/6 N, BDF1, and C3H/HeJ) were maintained under a 12:12 LD cycle of ~400 lux (light) to <1 lux red light (darkness), with lights on from 6 am to 6 pm. Ambient temperature was maintained at 21 ± 1 °C with a relative humidity of 30–70%. Food and water were available ad libitum. Both male and female mice aged 12 to 24 weeks were utilized for all in vivo experiments. Sex was not considered as a primary biological variable in the study design, as heritable antibody expression and susceptibility to *Borrelia* infection are not expected to be sex-dependent; therefore, data were aggregated across both sexes for final analysis.

### Testing mouse serum for full-length LA-2

Serum samples were obtained from mice by collecting blood in BD Microtainer serum separation tubes. ELISA plates were coated with rOspA at 5 µg/mL. Serum samples were applied to the coated plates, and LA-2 antibody levels were detected using the IgG ELISA detection kit (Bethyl Laboratories, E99-131). As a standard, purified LA-2 IgG2a (produced by GenScript) was used for quantification of circulating antibody levels at a starting concentration of 70 ng/mL.

### Infected tick challenges

*Borrelia burgdorferi*-infected nymphs used for challenges were prepared by feeding larval *Ixodes dammini* (Tufts colony) on mice (*Peromyscus leucopus* or *Mus musculus*) infected with the low-passage N40 strain (wild type). This strain is routinely maintained via tick-mouse-tick transfer, wherein uninfected larvae feed on infected mice, molt, and are subsequently used to infect naïve mice to perpetuate the in vivo cycle. At each challenge, all mice were infected from a single vial of infected nymphs with 70% infection rate, as determined by indirect immunofluorescence. Mice were bled for serum prior to infestation. At infestation, mice were anesthetized with ketamine/xylazine, and 10 nymphs were applied to the ears and nape of the neck of each mouse, which was held with a wire restraining tube loosely wrapped with a paper towel. Mice were liberated into standard shoebox cages held within a larger cage containing an inch of water, and provided rodent chow and water ad libitum. Engorged nymphs were collected from the water at 4-6 days after infestation, and stored in standard tick vials at 21 °C and 95% RH for 14 days when they were analyzed for evidence of infection.

### Bio-layer interferometry (BLI) assay

A full-length LA-2 IgG2a plasmid was constructed by GenScript. To generate LA-2 scFv-Fc, heavy chain and light chain variable region genes of LA-2 were amplified from the full-length plasmid and linked with a flexible (G4S)3 linker; the generated scFv was cloned into a modified pcDNA3.4 vector (Thermo Fisher Scientific). LA-2 IgG2a and LA-2 scFv-Fc plasmid were separately transfected into Expi293F cells (Thermo Fisher Scientific, catalog #A14635) following the standard ExpiFectamine293 protocol. The supernatants were harvested and incubated with Protein A-Sepharose 4B resin (Invitrogen) for 3 h at 4 °C followed by purification via gravity flow columns (BioRad) and buffer exchanged by centrifugation in Amicon centrifugal filters. Kinetic analysis was performed using an Octet Red96 instrument following the manufacturer’s instructions. Briefly, biotinylated OspA proteins were immobilized on streptavidin (SA) biosensors. The antigen-immobilized SA biosensors were then dipped into wells containing serially diluted (3.7–300 nM) antibody samples for 180 s for association. The sensors were then dipped into a kinetic buffer (PBST supplemented with 0.1% bovine serum albumin) for a 600 s dissociation step. A naked sensor was used as a non-specific binding control. Octet data analysis software (version 10.0.0.5) was used for kinetic curve fitting using the global fitting method.

### Linker sequence testing

Fifteen distinct linker sequences were evaluated to optimize expression, stability, and binding of the LA-2 scFv-Fc construct. Fifteen expression constructs were created, each with a different linker between the heavy and light chains of the antibody.

Lenti-X 293 T cells (Takara Bio, Cat# 632180) were transfected with the linker constructs using polyethylenimine (PEI) according to standard transfection protocols. Seventy-two hours post-transfection, supernatants were harvested for analysis. Antibody concentration was quantified using a mouse IgG2b ELISA kit (Bethyl Laboratories, E99-109). Antigen binding was assessed by coating plates with 5 µg/mL of rOspA (produced by GenScript), followed by detection using the same IgG2b ELISA kit. Full-length LA-2 IgG2b (produced by GenScript) was used as a standard for quantification at a starting concentration of 250 ng/mL, and as a positive control in the binding assay at a starting concentration of 1 µg/mL.

### Generation of LA-2 scFv-Albumin expressing *Mus musculus*

Pronuclear injections were conducted at MIT’s DCM Transgenics Core. C57BL/6N mouse embryos (generated by the MIT DCM Transgenics Core, originating from Charles River Laboratories) were microinjected with SpCas9, guide RNA (sequence: ACTCCAGTCTTTCTAGAAGA), and an LA-2 scFv-albumin expression cassette targeted to the *Rosa26* locus. The expression cassette contained a CAG promoter driving the expression of LA-2 scFv fused to mouse albumin, with a C-terminal His-tag for detection and purification. Injected embryos were implanted into pseudopregnant females. Of the 18 pups born, Sanger sequencing of PCR amplicons confirmed correct integration at the *Rosa26* locus in one pup.

### Genotyping LA-2 scFv-Albumin expressing *Mus musculus*

Genomic DNA was extracted from ear punches of LA-2 scFv-albumin expressing *Mus musculus* using the GenElute Mammalian Genomic DNA Mini-prep Kit (Sigma-Aldrich) according to the manufacturer’s instructions. Genotyping was performed by PCR using PrimeSTAR® DNA Polymerase (Takara Bio) with two primer sets. The first set targeted the wild-type *Rosa26* locus, utilizing *Rosa26*-F (5’-CTCTGAGTTGTTATCAGTAAGGGAGCTG-3’) and *Rosa26*-R (5’-CCTCCCATTTTCCTTATTTGCCCCTATTA-3’). The second set amplified the LA-2 scFv transgene using LA-2-scFv-F (5’-AAGGTCTCAAAAGAATGGGTTGGATCAAT-3’) and LA-2-scFv-R (5’-GGTTGAAGTGTGCTGGTGTAGTGTATAAG-3’). PCR products were analyzed by agarose gel electrophoresis to assess zygosity. Heterozygous mice showed amplification from both the *Rosa26* and LA-2 scFv primers, while homozygous mice displayed amplification only of the LA-2 scFv transgene.

### Testing mouse serum for LA-2 scFv-Albumin

Serum samples were collected from mice using BD Microtainer serum separation tubes. ELISA plates were coated with rOspA (produced by GenScript) at a concentration of 5 µg/mL. After applying the serum samples to the coated plates, LA-2 scFv-Albumin levels were detected using the Mouse Albumin ELISA Kit (Bethyl Laboratories, E99-134) with an anti-albumin secondary antibody. As a standard, purified LA-2 scFv-Albumin-His (produced by GenScript) was used for quantification of circulating antibody levels at a starting concentration of 30 µg/mL.

### Size-exclusion chromatography (SEC-HPLC)

Purified LA-2 scFv–albumin was analyzed by size-exclusion chromatography using a TSKgel G3000SWxl column (Tosoh Bioscience) on an HPLC system (Agilent 1260). Samples (47 µL) were injected and eluted isocratically using 0.1 M Na₂SO₄ in 0.118 M phosphate buffer (pH 6.7 ± 0.3) as the mobile phase. Elution was monitored by UV absorbance at 280 nm, and chromatograms were analyzed using standard Agilent 1260 HPLC system software. Molecular weight standards were run under identical conditions.

### Tick infection assay

Engorged nymphs were held for 14 days at 21 °C and 95% RH, and homogenized in 75 μL of PBS in microfuge tubes. The tubes were briefly centrifuged to pellet gross debris, 7.5 μL of the supernatant from each tick was applied to slides (Cel-Line 30-968, HTC), and allowed to dry before fixation in 100% acetone for 10 minutes. Slides were stained by indirect immunofluorescence using a rabbit polyclonal immune serum against *B. burgdorferi s.l*.; bound antibody was detected by staining with Alexa Fluor 488 conjugated anti-rabbit IgG. All slides were examined using epifluorescence at X400, and individual ticks were scored solely for the presence or absence of spirochetes.

### Mouse infection assay

Mice challenged with infected ticks were held for 21 days and bled for serum, which was analyzed for evidence of *B. burgdorferi* specific IgG antibody using the C6 peptide assay as described^21^, except that an endpoint EIA was used instead of a kinetic EIA. Briefly, biotinylated C6 peptide was bound to avidin-coated wells of a flat-bottomed microplate (Immulon 2), blocked with 3% fish gelatin in TBS (TBS-G), and 1:100 dilutions of mouse sera in TBS-G were incubated in duplicate for 1 hour at 37 °C. Bound antibody was detected using AP-goat anti-mouse IgG (gamma chain specific, Sigma), with a p-nitrophenyl phosphate substrate. A minimum of 6 negative control sera (uninfected B6 or C3H sera) optical densities were analyzed and used to calculate a cutoff value (mean + 3 standard deviations of the negative controls). Antibody to C6 is a sensitive indicator of *B. burgdorferi* infection.

### Xenodiagnosis

To assess the ability of mice to transmit *Borrelia burgdorferi* to feeding ticks, xenodiagnosis was performed^27^. At day 21 after the infected tick challenge, mice were infested with larval I. dammini ticks (Tufts colony), which were allowed to engorge. Engorged larvae were collected, placed into standard tick vials, and held at 21 °C and 95% RH until they molted 4–5 weeks later. After molting and hardening, samples from each vial were dissected, and guts smeared onto slides. The slides were dried, fixed in acetone, and stained by indirect immunofluorescence using an immune rabbit polyclonal serum against *B. burgdorferi s.l*. with secondary antibody comprising AlexaFluor 488 goat anti-rabbit IgG. Slides were examined for a minimum of 50 fields (X320) before declaring a negative. Any tick containing spirochetes (e.g., even 1 of 5 ticks) is evidence that the mouse is infected and is infectious.

### Statistics & Reproducibility

No statistical method was used to predetermine sample size. For initial in vitro construct optimization and screening, experiments were performed with 1 to 2 independent biological replicates to identify the optimal elements for subsequent rigorous in vivo validation. Sample sizes for the in vivo challenges were determined based on the number of viable transgenic founders generated from pronuclear injections and the subsequent availability of offspring across filial generations. These sample sizes proved sufficient to achieve high statistical significance in the primary endpoints. No data were excluded from the analyses.

The experiments were not randomized. Mice were allocated into experimental groups based on confirmation of the antibody gene insertion and zygosity (homozygous, heterozygous, or wild-type). To control for covariates during the infection assay, all mice within a given challenge cohort were exposed to infected nymphs derived from the same single vial to ensure a uniform tick infection rate.

Investigators performing the in vivo tick challenges, as well as the researcher scoring the immunofluorescence assays for tick infection, were blinded to the specific genotype of the mice. Unblinding was performed by a separate researcher only during the final data analysis phase. Blinding was not performed for in vitro biochemical and molecular assays (e.g., ELISA, SEC-HPLC, BLI), as these experiments rely on objective, automated quantitative readouts rather than subjective human scoring.

General statistical analyses for ELISA results and tick infection fractions were conducted using R (version 4.4.1). Statistical significance between groups was determined using unadjusted, two-sided Welch’s t-tests to account for unequal variances among independent biological replicates. Experimental findings were highly reproducible; transgene expression and heritability were independently verified across multiple consecutive breeding generations. All attempts at replicating the expression and infection assay findings across these independent generations were successful.

### Reporting summary

Further information on research design is available in the Nature Portfolio Reporting Summary linked to this article.

## Supplementary information

Supplementary Information

Reporting Summary

Transparent Peer Review file

## Source data

Source Data

## Data Availability

The data generated in this study have been deposited in the Mendeley Data repository under accession code 10.17632/wh65sf47bs.3 [10.17632/wh65sf47bs.3]^40^. Additionally, source data are provided with this paper. Materials Availability: All newly generated plasmids and engineered cell lines from this study are available from the corresponding authors upon request. The engineered *Mus musculus* lines generated in this study are also available from the corresponding authors upon request (note that certain lines are maintained only as finite stocks of cryopreserved sperm), subject to a standard Material Transfer Agreement (MTA). Requests for resources and reagents should be directed to Joanna Buchthal (buchthal@mit.edu) or Kevin Esvelt (esvelt@mit.edu). Authors will aim to process all initial requests for materials within 4 weeks, though the physical shipment of live mice or cryopreserved sperm is contingent upon the execution of the MTA and the coordination of specialized shipping logistics. Source data are provided with this paper.

## References

[1] Radolf, J. D., Caimano, M. J., Stevenson, B. & Hu, L. T. Of ticks, mice and men: understanding the dual-host lifestyle of Lyme disease spirochaetes. *Nat. Rev. Microbiol.* **10**, 87–99 (2012). 22230951 10.1038/nrmicro2714 PMC3313462

[2] Bunikis, J. et al. Borrelia burgdorferi infection in a natural population of Peromyscus Leucopus mice: a longitudinal study in an area where Lyme Borreliosis is highly endemic. *J. Infect. Dis.* **189**, 1515–1523 (2004). 15073690 10.1086/382594

[3] Lindsø, L. K., Viljugrein, H. & Mysterud, A. Vector competence of Ixodes ricinus instars for the transmission of Borrelia burgdorferi sensu lato in different small mammalian hosts. *Parasit. Vectors* **17**, 23 (2024). 38238796 10.1186/s13071-023-06110-7 PMC10797980

[4] Zhang, F., Gong, Z., Zhang, J. & Liu, Z. Prevalence of Borrelia burgdorferi sensu lato in rodents from Gansu, northwestern China. *BMC Microbiol.* **10**, 157 (2010). 20509909 10.1186/1471-2180-10-157 PMC2889951

[5] Frandsen, F., Bresciani, J. & Hansen, H. G. Prevalence of antibodies to Borrelia burgdorferi in Danish rodents. *APMIS* **103**, 247–253 (1995). 7612254

[6] Deblinger, R. D. & Rimmer, D. W. Efficacy of a permethrin-based acaricide to reduce the abundance of Ixodes dammini (Acari: Ixodidae). *J. Med. Entomol.* **28**, 708–711 (1991). 1941940 10.1093/jmedent/28.5.708

[7] Stafford, K. C. 3rd. Effectiveness of host-targeted permethrin in the control of Ixodes dammini (Acari: Ixodidae). *J. Med. Entomol.* **28**, 611–617 (1991). 1941927 10.1093/jmedent/28.5.611

[8] Fikrig, E. et al. Elimination of Borrelia burgdorferi from vector ticks feeding on OspA-immunized mice. *Proc. Natl. Acad. Sci. USA.* **89**, 5418–5421 (1992). 1608951 10.1073/pnas.89.12.5418 PMC49303

[9] Tsao, J. I. et al. An ecological approach to preventing human infection: vaccinating wild mouse reservoirs intervenes in the Lyme disease cycle. *Proc. Natl. Acad. Sci. USA.* **101**, 18159–18164 (2004). 15608069 10.1073/pnas.0405763102 PMC536054

[10] Fikrig, E., Barthold, S. W., Kantor, F. S. & Flavell, R. A. Protection of mice from Lyme borreliosis by oral vaccination with Escherichia coli expressing OspA. *J. Infect. Dis.* **164**, 1224–1227 (1991). 1955724 10.1093/infdis/164.6.1224

[11] Richer, L. M. et al. Reservoir targeted vaccine against Borrelia burgdorferi: a new strategy to prevent Lyme disease transmission. *J. Infect. Dis.* **209**, 1972–1980 (2014). 24523510 10.1093/infdis/jiu005 PMC4038139

[12] Hammond, A. et al. A CRISPR-Cas9 gene drive system targeting female reproduction in the malaria mosquito vector Anopheles gambiae. *Nat. Biotechnol.* **34**, 78–83 (2016). 26641531 10.1038/nbt.3439 PMC4913862

[13] Hoermann, A. et al. Gene drive mosquitoes can aid malaria elimination by retarding Plasmodium sporogonic development. *Sci. Adv.* **8**, eabo1733 (2022). 36129981 10.1126/sciadv.abo1733 PMC9491717

[14] Jakobovits, A., Amado, R. G., Yang, X., Roskos, L. & Schwab, G. From XenoMouse technology to panitumumab, the first fully human antibody product from transgenic mice. *Nat. Biotechnol.* **25**, 1134–1143 (2007). 17921999 10.1038/nbt1337

[15] Peterson, N. C. Advances in monoclonal antibody technology: genetic engineering of mice, cells, and immunoglobulins. *ILAR J.* **46**, 314–319 (2005). 15953839 10.1093/ilar.46.3.314

[16] Castilla, J., Pintado, B., Sola, I., Sánchez-Morgado, J. M. & Enjuanes, L. Engineering passive immunity in transgenic mice secreting virus-neutralizing antibodies in milk. *Nat. Biotechnol.* **16**, 349–354 (1998). 9555725 10.1038/nbt0498-349 PMC7097410

[17] Schaible, U. E. et al. Monoclonal antibodies specific for the outer surface protein A (OspA) of Borrelia burgdorferi prevent Lyme borreliosis in severe combined immunodeficiency (scid) mice. *Proc. Natl. Acad. Sci. USA.* **87**, 3768–3772 (1990). 2339119 10.1073/pnas.87.10.3768 PMC53984

[18] Ding, W. et al. Structural identification of a key protective B-cell epitope in Lyme disease antigen OspA. *J. Mol. Biol.* **302**, 1153–1164 (2000). 11183781 10.1006/jmbi.2000.4119

[19] Wang, Y. et al. Pre-exposure Prophylaxis with OspA-specific human monoclonal antibodies protects mice against tick transmission of lyme disease spirochetes. *J. Infect. Dis.* **214**, 205–211 (2016). 27338767 10.1093/infdis/jiw151 PMC4918831

[20] Pinkert, C. A., Ornitz, D. M., Brinster, R. L. & Palmiter, R. D. An albumin enhancer located 10 kb upstream functions along with its promoter to direct efficient, liver-specific expression in transgenic mice. *Genes Dev.* **1**, 268–276 (1987). 3678824 10.1101/gad.1.3.268

[21] Bacon, R. M. et al. Serodiagnosis of Lyme disease by kinetic enzyme-linked immunosorbent assay using recombinant VlsE1 or peptide antigens of Borrelia burgdorferi compared with 2-tiered testing using whole-cell lysates. *J. Infect. Dis.* **187**, 1187–1199 (2003). 12695997 10.1086/374395 PMC7109709

[22] Tao, H.-Y., Wang, R.-Q., Sheng, W.-J. & Zhen, Y.-S. The development of human serum albumin-based drugs and relevant fusion proteins for cancer therapy. *Int. J. Biol. Macromol.* **187**, 24–34 (2021). 34284054 10.1016/j.ijbiomac.2021.07.080

[23] Marsh, M. C. & Owen, S. C. Therapeutic fusion proteins. *AAPS J.* **26**, 3 (2023). 38036919 10.1208/s12248-023-00873-8

[24] Gu, B., Posfai, E. & Rossant, J. Efficient generation of targeted large insertions by microinjection into two-cell-stage mouse embryos. *Nat. Biotechnol.* **36**, 632–637 (2018). 29889212 10.1038/nbt.4166

[25] Iii, S. R. T. & Goethert, H. K. Perpetuation of Borreliae. *Curr. Issues Mol. Biol.* **42**, 267–306 (2021). 33300495 10.21775/cimb.042.267 PMC8299828

[26] Snow, A. A. et al. Tick densities and infection prevalence on coastal islands in Massachusetts, USA: Establishing a baseline. *Insects* **14**, 628 (2023). 37504634 10.3390/insects14070628 PMC10380421

[27] Marques, A. et al. Xenodiagnosis to detect Borrelia burgdorferi infection: a first-in-human study. *Clin. Infect. Dis.* **58**, 937–945 (2014). 24523212 10.1093/cid/cit939 PMC3952603

[28] Buchthal, J., Evans, S. W., Lunshof, J., Telford, S. R. 3rd & Esvelt, K. M. Mice Against Ticks: an experimental community-guided effort to prevent tick-borne disease by altering the shared environment. *Philos. Trans. R. Soc. Lond. B Biol. Sci.* **374**, 20180105 (2019). 30905296 10.1098/rstb.2018.0105 PMC6452264

[29] Mills, J. N., Ksiazek, T. G., Peters, C. J. & Childs, J. E. Long-term studies of hantavirus reservoir populations in the southwestern United States: a synthesis. *Emerg. Infect. Dis.* **5**, 135–142 (1999). 10081681 10.3201/eid0501.990116 PMC2627702

[30] Gomes-Solecki, M., Santecchia, I. & Werts, C. Animal models of leptospirosis: Of mice and hamsters. *Front. Immunol.* **8**, 58 (2017). 28270811 10.3389/fimmu.2017.00058 PMC5318464

[31] Smither, A. R. et al. Novel tools for Lassa virus surveillance in Peri-domestic rodents. *medRxiv*10.1101/2023.03.17.23287380 (2023).

[32] Schwan, T. G., Piesman, J., Golde, W. T., Dolan, M. C. & Rosa, P. A. Induction of an outer surface protein on Borrelia burgdorferi during tick feeding. *Proc. Natl. Acad. Sci. USA*. **92**, 2909–2913 (1995). 7708747 10.1073/pnas.92.7.2909 PMC42328

[33] de Silva, A. M. et al. Influence of outer surface protein A antibody on Borrelia burgdorferi within feeding ticks. *Infect. Immun.* **67**, 30–35 (1999). 9864192 10.1128/iai.67.1.30-35.1999 PMC96273

[34] Bhattacharyya, A. & Mantis, N. J. OspA antibodies inhibit the in vitro transmigration of *Borreliellia burgdorferi*. *bioRxiv*10.1101/2025.11.20.689610 (2025).

[35] Dunham-Ems, S. M. et al. Live imaging reveals a biphasic mode of dissemination of Borrelia burgdorferi within ticks. *J. Clin. Invest.* **119**, 3652–3665 (2009). 19920352 10.1172/JCI39401 PMC2786795

[36] Buchthal, J. et al. Non-invasive ovulation tracking enables genetic engineering in wild rodents. *Cell Rep. Methods.* **6**, 10.1016/j.crmeth.2026.101311 (2026). 10.1016/j.crmeth.2026.101311 PMC12946743 41709464

[37] Goethert, H., O’Callahan, A., Johnson, R., Roden-Reynolds, P. & Telford, S. Minor hosts have a major impact on the enzootic transmission of Borrelia burgdorferi. *Am. J. Trop. Med. Hyg*. 10.4269/ajtmh.24-0283 (2024). 10.4269/ajtmh.24-0283 PMC11720800 39531726

[38] Goundie, T. R. & Vessey, S. H. Survival and dispersal of young white-footed mice born in nest boxes. *J. Mammal.* **67**, 53–60 (1986).

[39] Postic, C. et al. Dual roles for glucokinase in glucose homeostasis as determined by liver and pancreatic beta cell-specific gene knock-outs using Cre recombinase. *J. Biol. Chem.* **274**, 305–315 (1999). 9867845 10.1074/jbc.274.1.305

[40] Buchthal, J. et al. Heritable immunization of mice against Lyme disease enables ecological disease prevention. Mendeley Data 10.17632/wh65sf47bs.3 (2026). 10.1038/s41467-026-71757-6 PMC13328618 42049716

