# Supplementary material for: Heritable immunization of mice against Lyme disease enables ecological disease prevention
Source: Nat Commun. 2026 Apr 28;17:5814. doi: 10.1038/s41467-026-71757-6 (PMC13328618; doi:10.1038/s41467-026-71757-6)
Supplement: Supplementary file 1 — Supplementary Information [file 41467_2026_71757_MOESM1_ESM.pdf]

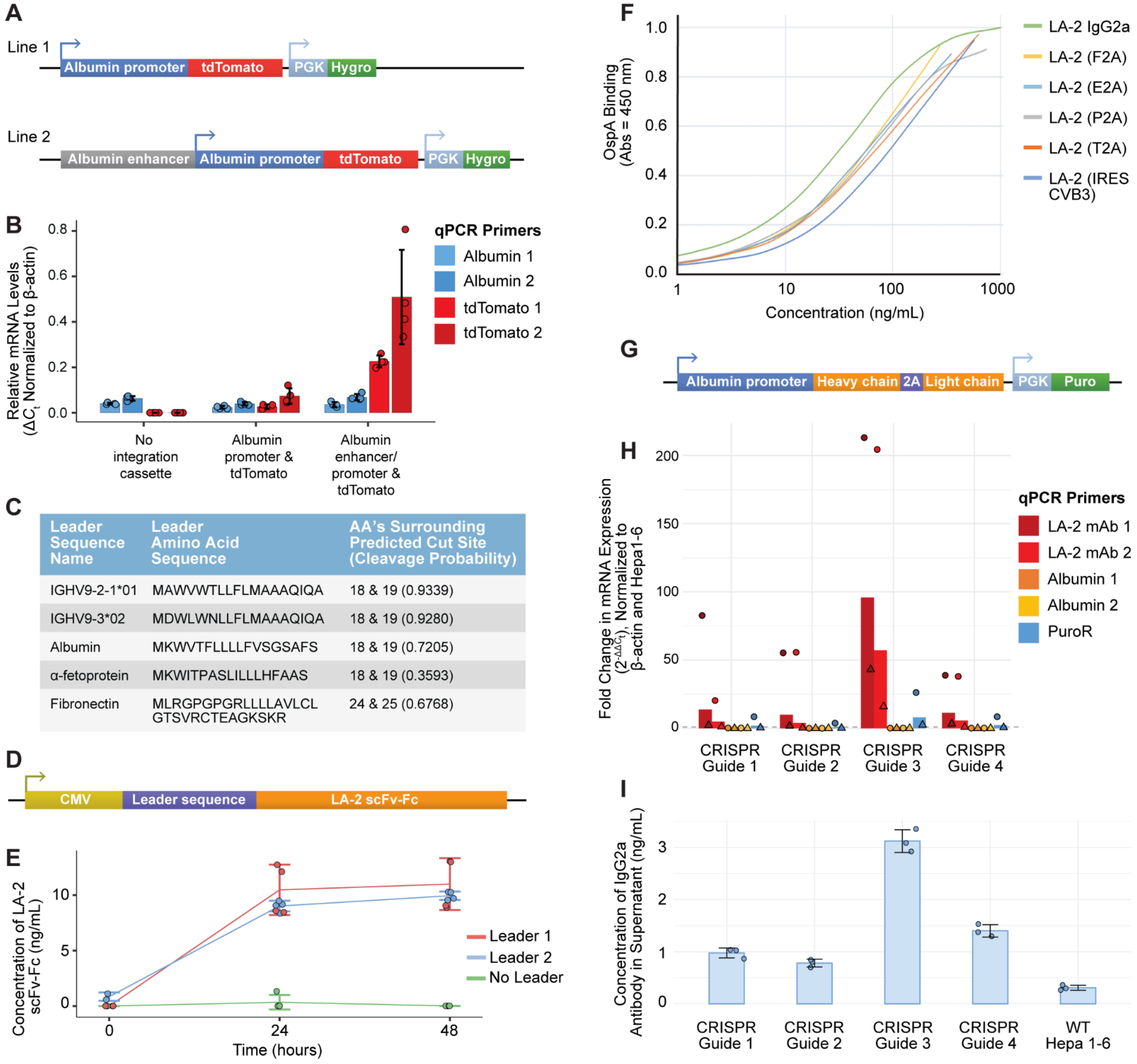

Leader Sequence Name	Leader Amino Acid Sequence	AA's Surrounding Predicted Cut Site (Cleavage Probability)
IGHV9-2-1*01	MAWVWTLLFLMAAAQIQA	18 & 19 (0.9339)
IGHV9-3*02	MDWLWNLLFLMAAAQIQA	18 & 19 (0.9280)
Albumin	MKWVTFLLLLFVSGSAFS	18 & 19 (0.7205)
α-fetoprotein	MKWITPASLILLLHFAAS	18 & 19 (0.3593)
Fibronectin	MLRGPGPGRLLLLAVLCL GTSVRCTEAGKSKR	24 & 25 (0.6768)

Supplementary figure 1. Optimization and Validation of LA-2 Antibody Expression *in vitro*

A) Schematic representation of the CRISPR-modified cell lines used for testing albumin expression machinery, incorporating the minimal albumin promoter with and without the albumin enhancer. **B)** RT-qPCR analysis showing relative mRNA levels of tdTomato and albumin in cells transfected with the constructs described in (A). Data represent *n* = 2 independent biological replicates, with 4 technical replicates per biological replicate. **C)** Leader sequences evaluated for their ability to direct LA-2 antibody secretion, including sequences from mouse albumin, alpha-fetoprotein, and fibronectin, along with their predicted cleavage efficiency. **D)** Schematic of the LA-2 scFv-Fc expression construct under the control of a CMV promoter, including the leader sequences tested for secretion efficiency. **E)** ELISA results comparing LA-2 scFv-Fc concentrations in the supernatant of cells transfected with constructs containing two different leader sequences, measured over a 48-hour period (*n* = 2 independent biological replicates, measured with 2 technical replicates per biological replicate at 24 and 48 hours, and a single measurement at 0 hours). **F)** OspA binding activity of LA-2 antibodies produced using different bicistronic elements, measured by ELISA (*n* = 2 independent biological replicates). **G)** Schematic of CRISPR-modified cell lines expressing the full-length LA-2 antibody. **H)** RT-qPCR analysis of LA-2 mRNA expression in engineered cells, normalized to β-actin and Hepa1-6. Circles and triangles represent *n* = 2 independent biological replicates, each measured with 2 technical replicates (representing distinct primer sets); the dashed line denotes the Hepa1-6 reference. **I)** Concentration of IgG2a in the supernatant of CRISPR-modified cells, quantified by ELISA (*n* = 3 technical replicates from a single biological transfection). Error bars represent mean ± SD. Source data are provided as a Source Data file.

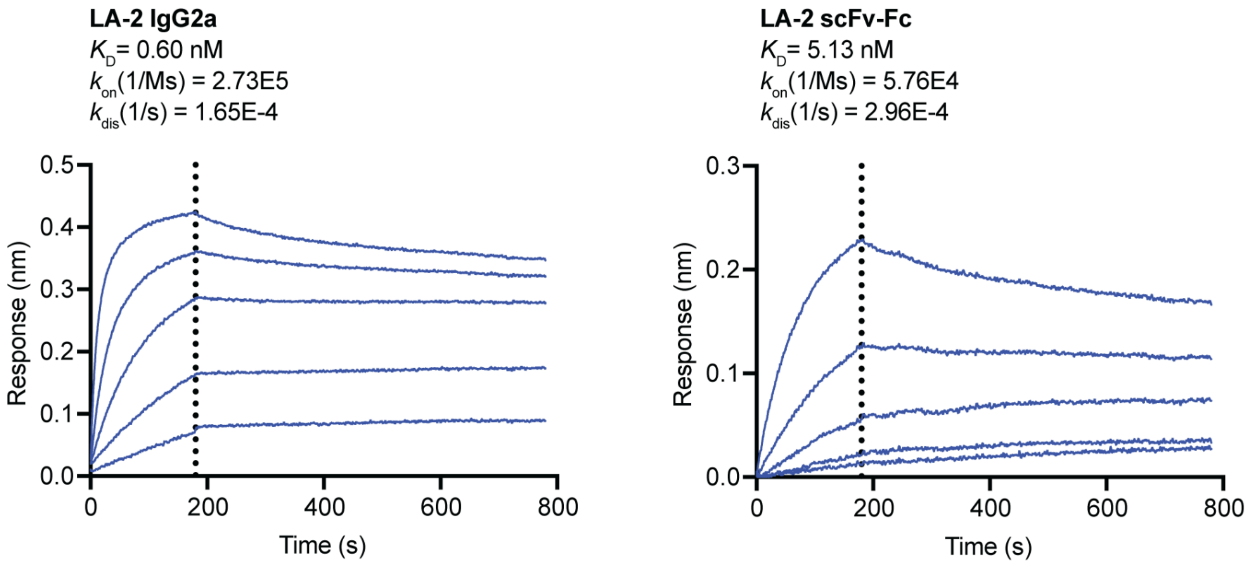

Supplementary figure 2. Binding Affinity of LA-2 IgG2a and LA-2 scFv
Binding affinity data for LA-2 IgG2a and its scFv counterpart with a Vh-(G4S)3-Vl linker. Association rate constant (k_{on}), dissociation rate constant (k_{dis}), and equilibrium dissociation constant (K_D) values are reported.

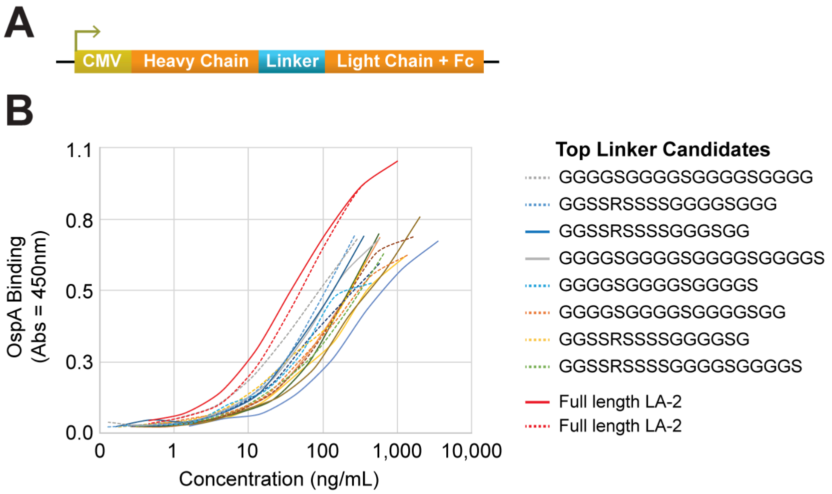

Supplementary figure 3. Evaluation of Linker Sequences for Optimizing LA-2 scFv-Fc
A) Schematic representation of the expression construct used to evaluate different linker sequences between the LA-2 scFv heavy and light chains. The construct includes a CMV promoter driving the expression of the LA-2 scFv-Fc fusion protein, with various linkers designed to modulate flexibility and length. **B)** OspA binding data from ELISA assays comparing the performance of different linker sequences in Lenti-X 293T cells 72 hours post-transfection (n = 1 independent biological replicate per linker). The binding profiles of the top linker candidates were assessed against purified full-length LA-2 antibody (red lines).. Source data are provided as a Source Data file.

A

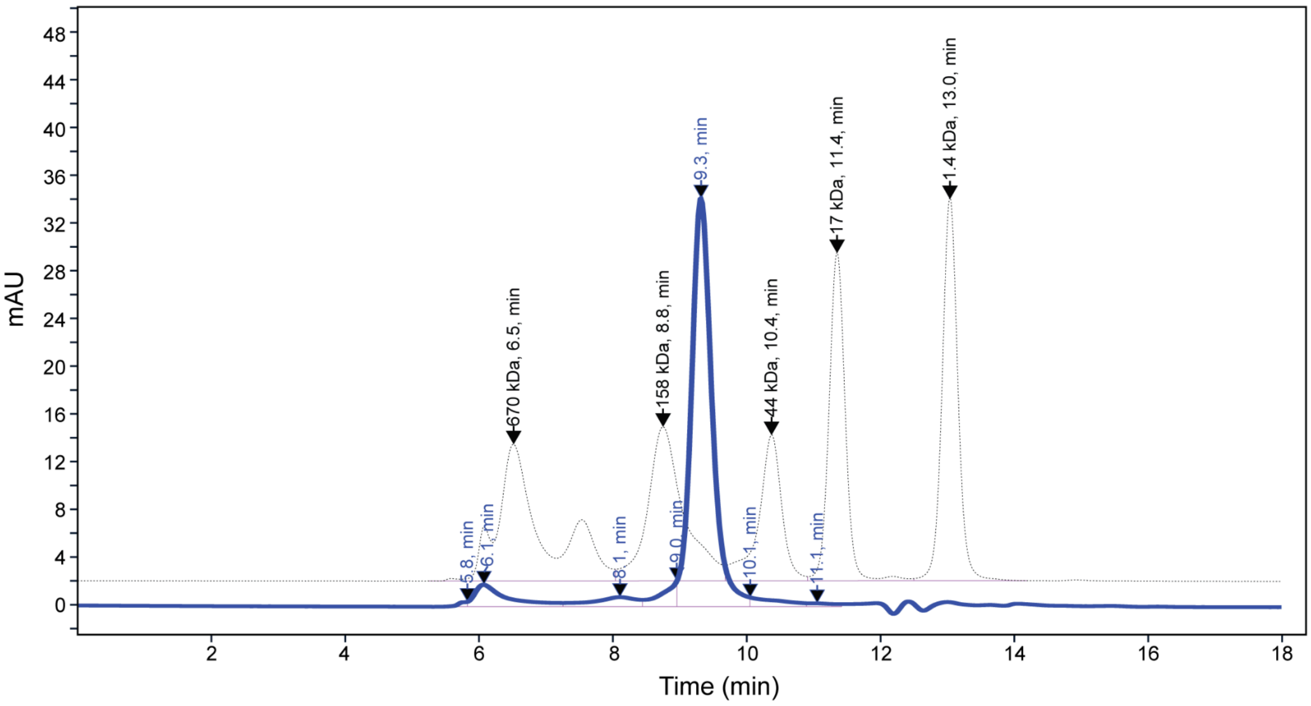

B

RT (min)	Height	Width (min)	Area	Area %
5.8	0.4	0.7	3.5	0
6.1	1.8	1.4	64.7	7
8.1	0.8	1.2	37.7	4
9.0	2.1	0.5	32.7	4
9.3	34.2	1.1	740.2	81
10.1	0.8	0.8	24.6	3
11.1	0.3	0.5	7.7	1
		Sum	911.12	

Supplementary figure 4. Size-exclusion chromatography of LA-2 scFv–albumin
A) Size-exclusion chromatography (SEC) profile of purified LA-2 scFv–albumin. **B)** Summary table corresponding to the chromatography profile shown in (A).
