# Supplementary material for: Heritable immunization of mice against Lyme disease enables ecological disease prevention
Source: Nat Commun. 2026 Apr 28;17:5814. doi: 10.1038/s41467-026-71757-6 (PMC13328618; doi:10.1038/s41467-026-71757-6)
Supplement: Supplementary file 2 — Reporting Summary [file 41467_2026_71757_MOESM2_ESM.pdf]

nature portfolio

Corresponding author(s): Kevin M. Esvelt

Last updated by author(s): March 26, 2026

Reporting Summary

Nature Portfolio wishes to improve the reproducibility of the work that we publish. This form provides structure for consistency and transparency in reporting. For further information on Nature Portfolio policies, see our Editorial Policies and the Editorial Policy Checklist.

Statistics

For all statistical analyses, confirm that the following items are present in the figure legend, table legend, main text, or Methods section.

n/a	Confirmed	
[ ]	[x]	The exact sample size (*n*) for each experimental group/condition, given as a discrete number and unit of measurement
[ ]	[x]	A statement on whether measurements were taken from distinct samples or whether the same sample was measured repeatedly
[ ]	[x]	The statistical test(s) used AND whether they are one- or two-sided<br>*Only common tests should be described solely by name; describe more complex techniques in the Methods section.*
[x]	[ ]	A description of all covariates tested
[ ]	[x]	A description of any assumptions or corrections, such as tests of normality and adjustment for multiple comparisons
[ ]	[x]	A full description of the statistical parameters including central tendency (e.g. means) or other basic estimates (e.g. regression coefficient) AND variation (e.g. standard deviation) or associated estimates of uncertainty (e.g. confidence intervals)
[ ]	[x]	For null hypothesis testing, the test statistic (e.g. *F*, *t*, *r*) with confidence intervals, effect sizes, degrees of freedom and *P* value noted<br>*Give P values as exact values whenever suitable.*
[x]	[ ]	For Bayesian analysis, information on the choice of priors and Markov chain Monte Carlo settings
[x]	[ ]	For hierarchical and complex designs, identification of the appropriate level for tests and full reporting of outcomes
[x]	[ ]	Estimates of effect sizes (e.g. Cohen's *d*, Pearson's *r*), indicating how they were calculated

Our web collection on statistics for biologists contains articles on many of the points above.

Software and code

Policy information about availability of computer code

Data collection	Data were collected using standard commercial instrument software. Bio-layer interferometry (BLI) data were acquired using the Octet Red96 instrument software. Size-exclusion chromatography data were collected using standard Agilent 1260 HPLC system software. No custom software or code was used for data collection.
Data analysis	Statistical analysis and data visualization were performed using R (version 4.4.1). Kinetic curve fitting was performed using Octet data analysis software (version 10.0.0.5). In silico analysis of leader sequences and cleavage efficiency utilized IMGT's V-Quest tool and SignalP software (version 5.0), respectively. Chromatograms were analyzed using standard Agilent 1260 HPLC system software. No custom algorithms or custom code were utilized.

For manuscripts utilizing custom algorithms or software that are central to the research but not yet described in published literature, software must be made available to editors and reviewers. We strongly encourage code deposition in a community repository (e.g. GitHub). See the Nature Portfolio guidelines for submitting code & software for further information.

Data

Policy information about availability of data

All manuscripts must include a data availability statement. This statement should provide the following information, where applicable:

- Accession codes, unique identifiers, or web links for publicly available datasets
- A description of any restrictions on data availability
- For clinical datasets or third party data, please ensure that the statement adheres to our policy

The data generated in this study have been deposited in the Mendeley Data repository under accession code 10.17632/wh65sf47bs.3 [https://doi.org/10.17632/wh65sf47bs.3].

Research involving human participants, their data, or biological material

Policy information about studies with human participants or human data. See also policy information about sex, gender (identity/presentation), and sexual orientation and race, ethnicity and racism.

Reporting on sex and gender	*Use the terms sex (biological attribute) and gender (shaped by social and cultural circumstances) carefully in order to avoid confusing both terms. Indicate if findings apply to only one sex or gender; describe whether sex and gender were considered in study design; whether sex and/or gender was determined based on self-reporting or assigned and methods used. Provide in the source data disaggregated sex and gender data, where this information has been collected, and if consent has been obtained for sharing of individual-level data; provide overall numbers in this Reporting Summary. Please state if this information has not been collected. Report sex- and gender-based analyses where performed, justify reasons for lack of sex- and gender-based analysis.*
Reporting on race, ethnicity, or other socially relevant groupings	*Please specify the socially constructed or socially relevant categorization variable(s) used in your manuscript and explain why they were used. Please note that such variables should not be used as proxies for other socially constructed/relevant variables (for example, race or ethnicity should not be used as a proxy for socioeconomic status). Provide clear definitions of the relevant terms used, how they were provided (by the participants/respondents, the researchers, or third parties), and the method(s) used to classify people into the different categories (e.g. self-report, census or administrative data, social media data, etc.) Please provide details about how you controlled for confounding variables in your analyses.*
Population characteristics	*Describe the covariate-relevant population characteristics of the human research participants (e.g. age, genotypic information, past and current diagnosis and treatment categories). If you filled out the behavioural & social sciences study design questions and have nothing to add here, write "See above."*
Recruitment	*Describe how participants were recruited. Outline any potential self-selection bias or other biases that may be present and how these are likely to impact results.*
Ethics oversight	*Identify the organization(s) that approved the study protocol.*

Note that full information on the approval of the study protocol must also be provided in the manuscript.

Field-specific reporting

Please select the one below that is the best fit for your research. If you are not sure, read the appropriate sections before making your selection.

[x] Life sciences [ ] Behavioural & social sciences [ ] Ecological, evolutionary & environmental sciences

For a reference copy of the document with all sections, see nature.com/documents/nr-reporting-summary-flat.pdf

Life sciences study design

All studies must disclose on these points even when the disclosure is negative.

Sample size	No statistical methods were used to predetermine sample sizes. Sample sizes for the in vivo challenges were determined based on the number of viable transgenic founders generated from pronuclear injections and the subsequent availability of offspring across different filial generations. These sample sizes proved sufficient to achieve high statistical significance in our primary endpoints (e.g., $P < 0.001$ for reduced infection).
Data exclusions	No data were excluded from the analyses.
Replication	Experimental findings were highly reproducible. Transgene expression and heritability were independently verified across multiple consecutive breeding generations (up to F4 for the full-length model and 6 generations for the scFv-albumin model). All attempts at replicating the expression and infection assay findings across these independent generations were successful.
Randomization	Organisms were allocated into experimental groups based on their confirmed genetic sequence and zygosity (homozygous, heterozygous, or wild-type) rather than by random assignment. To control for covariates during the infection assay, all mice within a given challenge cohort were exposed to infected nymphs derived from the exact same single vial to ensure a uniform tick infection rate.

Blinding	Investigators performing the in vivo tick challenges, as well as the researcher scoring the immunofluorescence assays for tick infection, were blinded to the specific genotype (homozygous, heterozygous, or wild-type) of the mice. Unblinding was performed by a separate researcher only during the final data analysis phase. Blinding was not performed for in vitro biochemical and molecular assays (e.g., ELISA, SEC-HPLC, BLI), as these experiments rely on objective, automated quantitative readouts rather than subjective human scoring.

Reporting for specific materials, systems and methods

We require information from authors about some types of materials, experimental systems and methods used in many studies. Here, indicate whether each material, system or method listed is relevant to your study. If you are not sure if a list item applies to your research, read the appropriate section before selecting a response.

Materials & experimental systems

n/a	Involved in the study
☐	☒ Antibodies
☐	☒ Eukaryotic cell lines
☒	☐ Palaeontology and archaeology
☐	☒ Animals and other organisms
☒	☐ Clinical data
☒	☐ Dual use research of concern
☒	☐ Plants

Methods

n/a	Involved in the study
☒	☐ ChIP-seq
☒	☐ Flow cytometry
☒	☐ MRI-based neuroimaging

Antibodies

Antibodies used	Commercial ELISA Kits (proprietary capture/detection antibodies):<br>- Mouse IgG2a ELISA Kit (Bethyl Laboratories, Cat# E99-107)<br>- Mouse IgG2b ELISA Kit (Bethyl Laboratories, Cat# E99-109)<br>- Mouse IgG ELISA Kit (Bethyl Laboratories, Cat# E99-131)<br>- Mouse Albumin ELISA Kit (Bethyl Laboratories, Cat# E99-134)<br><br>Custom/Recombinant Antibodies (used as standards or controls):<br>- Full-length LA-2 IgG2a (Produced by GenScript; working concentrations: 500 ng/mL and 70 ng/mL for quantification standards, 1 µg/mL for binding control)<br>- Full-length LA-2 IgG2b (Produced by GenScript; working concentrations: 250 ng/mL for quantification standard, 1 µg/mL for binding control)<br>- LA-2 scFv-Albumin-His (Produced by GenScript; working concentration: 30 µg/mL for quantification standard)<br><br>Immunofluorescence & Infection Assays:<br>- Goat anti-Rabbit IgG (H+L) Cross-Adsorbed Secondary Antibody, Alexa Fluor 488 (Life Technologies, Cat# A11008, Lot# 1348202; 1:2000 dilution)<br>- Goat anti-Mouse IgG (gamma-chain specific)–Alkaline Phosphatase (Sigma-Aldrich, Cat# A1047, Lot# 89F8820; 1:1000 dilution)<br>- Rabbit anti-Borrelia burgdorferi s.l. polyclonal serum (Custom, produced in-house by S. Telford; 1:100 dilution)
Validation	All commercial ELISA kits (Bethyl Laboratories) and commercial secondary antibodies are validated by their respective manufacturers for the specific binding and detection of mouse immunoglobulins, mouse albumin, or rabbit IgG, respectively, as described on the manufacturers' websites.<br>The custom recombinant LA-2 antibodies (IgG2a, IgG2b, and scFv-Albumin) generated by GenScript were validated in-house within this study for specific binding to recombinant outer surface protein A (rOspA) via ELISA and bio-layer interferometry (BLI), with binding kinetics and stability data provided in the main text and supplement.<br>The rabbit polyclonal immune serum against B. burgdorferi s.l. has been routinely validated for the detection of spirochetes in tick tissues via indirect immunofluorescence using established laboratory protocols.

Eukaryotic cell lines

Policy information about cell lines and Sex and Gender in Research

Cell line source(s)	Three established cell lines were utilized in this study:<br>• Hepa 1-6 cells were obtained from ATCC (Cat# CRL-1830)<br>• Expi293F cells were obtained from Thermo Fisher Scientific (Cat# A14635)<br>• Lenti-X 293T cells were obtained from Takara Bio (Cat# 632180)
Authentication	Cell lines were utilized as supplied by the manufacturer. None were independently authenticated in-house.
Mycoplasma contamination	The cell lines were not tested for mycoplasma contamination, as they were newly obtained from commercial vendors and utilized strictly for short-term, transient in vitro transfections to optimize construct design prior to in vivo use.

Commonly misidentified lines
(See ICLAC register)

No commonly misidentified cell lines (as listed in the ICLAC register) were used in this study.

Animals and other research organisms

Policy information about studies involving animals; ARRIVE guidelines recommended for reporting animal research, and Sex and Gender in Research

Laboratory animals	Mus musculus (substrains C57BL/6N, BDF1, and C3H/HeJ) aged 12 to 24 weeks were used in this study. C57BL/6N embryos and adult mice were sourced from Charles River Laboratories, either directly or through the MIT DCM Transgenics Core. BDF1 embryos utilized for pronuclear injections were generated in-house by the Whitehead Institute GEM Core by mating DBA/2N males with C57BL/6N females (both parental lines sourced from Charles River Laboratories). C3H/HeJ mice were sourced from The Jackson Laboratory. The study also utilized laboratory-reared Ixodes dammini ticks (larvae and nymphs) sourced from an established Tufts University colony, as well as laboratory mice infected with Borrelia burgdorferi (low-passage strain N40) for tick feeding. Mice were maintained under a 12:12 light/dark cycle (~400 lux light to <1 lux red light), an ambient temperature of 21 ± 1 °C, and 30–70% humidity, with food and water available ad libitum.
Wild animals	This study did not involve wild animals.
Reporting on sex	Both male and female mice were utilized for breeding and in vivo tick infection challenges. Sex was not considered as a primary biological variable in the study design for infection outcomes, as heritable antibody expression (driven by the minimal albumin or CAG promoters) and direct antibody-mediated spirochete neutralization are not expected to be sex-dependent. Consequently, sex-based analyses were not performed, and data were aggregated across both sexes for the final analysis.
Field-collected samples	This study did not involve samples collected from the field.
Ethics oversight	The Massachusetts Institute of Technology's Committee on Animal Care (CAC) approved all mouse procedures and all mice were maintained at MIT in strict accordance with institutional protocols, DOD guidelines, and the Guide for the Care and Use of Laboratory Animals. The Tufts University Institutional Animal Care & Use Committee (IACUC) approved all mouse studies performed at Tufts University.

Note that full information on the approval of the study protocol must also be provided in the manuscript.

Plants

Seed stocks	*Report on the source of all seed stocks or other plant material used. If applicable, state the seed stock centre and catalogue number. If plant specimens were collected from the field, describe the collection location, date and sampling procedures.*
Novel plant genotypes	*Describe the methods by which all novel plant genotypes were produced. This includes those generated by transgenic approaches, gene editing, chemical/radiation-based mutagenesis and hybridization. For transgenic lines, describe the transformation method, the number of independent lines analyzed and the generation upon which experiments were performed. For gene-edited lines, describe the editor used, the endogenous sequence targeted for editing, the targeting guide RNA sequence (if applicable) and how the editor was applied.*
Authentication	*Describe any authentication procedures for each seed stock used or novel genotype generated. Describe any experiments used to assess the effect of a mutation and, where applicable, how potential secondary effects (e.g. second site T-DNA insertions, mosiacism, off-target gene editing) were examined.*
