# Supplementary material for: Heritable immunization of mice against Lyme disease enables ecological disease prevention
Source: Nat Commun. 2026 Apr 28;17:5814. doi: 10.1038/s41467-026-71757-6 (PMC13328618; doi:10.1038/s41467-026-71757-6)
Supplement: Supplementary file 3 — Transparent Peer Review file [file 41467_2026_71757_MOESM3_ESM.pdf]

nature portfolio

Peer Review File

Heritable immunization of mice against Lyme disease enables ecological disease prevention

Corresponding Author: Professor Kevin Esvelt

This file contains all reviewer reports in order by version, followed by all author rebuttals in order by version.

Version 0:

Reviewer comments:

Reviewer #1

(Remarks to the Author)
More than 30 years have elapsed since the first reports that immunization with outer surface protein A (OspA) confers protection against Lyme disease. While the large majority of subsequent studies have focused on the development of OspA as a vaccine for humans, there also have been efforts to immunize reservoir hosts (typically white footed mice in North America) in the wild to interrupt the enzootic cycle that sustains Lyme disease spirochetes in nature. All of these studies have employed strategies to administer OspA to mice as a vaccinogen. This tautly written, clearly laid out study by Buchthal et al. takes a radically different and highly innovative approach: it uses state-of-the-art genetic engineering to create a murine mouse strain that heritably produces substantial levels of a single chain variable fragment derived from a well characterized murine monoclonal antibody (LA2) directed against a protective OspA epitope. The authors demonstrate convincingly that the strategy has great promise. Transmission of B. burgdorferi by infected nymphs is greatly diminished as is the ability of infected, heritably immunized mice to pass the bacterium on to feeding larvae. Nevertheless, some aspects of this impressive study relating mostly to the borreliology could be clarified and/or strengthened:

1. Lines 154 and Suppl. Fig. 1B. The text states that the constructs with and without the albumin enhancer produced comparable levels of tdTomato with and without the albumin enhancer. However, the figure appears to show much greater levels of tdTomato RNA from the construct containing the albumin enhancer.

2. Lines 288-291 and Suppl Fig. 2. What were the sources of the antibodies used for these experiments?

3. Lines 358-383 and Figure 2D. The authors measure production of C6 antibodies as their indicator of transmission by feeding nymphs. However, this is a rather limited analysis. A more accurate picture of spirochete burdens could have been obtained by culture of ear punches and internal tissues as well as qPCR. The authors subsequently (lines 417-39) used xenodiagnosis to assess acquisition of spirochetes by feeding larvae. While acquisition was largely prevented, the results also showed that some mice were indeed infected, further arguing for a more robust characterization of burdens. Because OspA antibodies interfere with acquisition (as shown here), these results do not directly speak to the spirochete burdens in the mice upon which the larvae fed.

4. Lines 388-412 and Figure 2E. An important aspect of the paper is the effect of the secreted antibody fragment on spirochetes in infected, feeding nymphs. The authors anticipated that the antibody would be borreliacidal although that is not what they believe they observed. However, the data provided is not easily interpretable since they relied entirely upon IFA rather than quantitative culture of spirochetes from nymphal midguts. Moreover, the presentation of the IFA data is confusing. The Methods (lines 1028-1032) states that they graded the IFAs on a scale of 0-4. The Results, on the other hand, states that “each tick was categorized based on the presence or absence of spirochetes regardless of infection intensity”, while Figure 2E shows, according to the labeling of the Y axis, “the fraction of infected ticks per challenged mouse”. The wording here is obscure, but it does not indicate quantitation. The explanation of the results provided in the Discussion (lines 476-487) further adds to the confusion because it largely centers on older data relating to reciprocal expression of OspA and OspC rather than more recent studies of how spirochetes migrate through the midgut. Along these lines, it should be noted that the mechanism whereby OspA antibodies prevent transmission is far from clear; not all investigators find that OspA antibodies, including LA2, are borreliacidal.

5. The Discussion of next steps is candid and thoughtful. However, the statement “Our recent advances in rodent ovulation tracking…” (lines 521-524) needs clarification.

Reviewer #2

(Remarks to the Author)
The report ¨Heritable immunization of mice against Lyme disease enables ecological disease prevention¨ by a large group led by Joanna Buchthal describes the creation and the characterization of a single chain antibody (scFv) to Borrelia burgdorferi that was engineered into laboratory mice. Several generations of engineered mice produced scFv’s capable of binding the parent antigen (OspA) and protecting mice against experimental infection via tick bites. This report clearly demonstrates the feasibility of creating heritable immunization – a preamble to multiple applications. This report also provides a step by step description of the creation of the full-length antibody expression in mice that did not produce appreciable immunity, and was thus abandoned. By any measure, this report is a robust feat of genetics with far-reaching possibilities. The authors should be commended for not overstating the field (disease control) applications of their work as these can present both foreseen and unforeseen challenges.
The format of this manuscript leads to some issues of readability. One aspect deals with the negative (but important) aspects of engineering a full-length antibody. This manuscript first delves into the generation and validation of liver-specific full-length LA-2 antibody expressing mice. This part of the manuscript comprises the supplemental figure data as well as data in Figure 1. As mentioned, the mice engineered with full length antibody showed erratic production of the antibody as well as a response to challenge. Hence, the effort of creating mice expressing a full-length antibody provided a negative result. Yet, the methodology for its creation occupies a large amount (one half) of space in the manuscript and no mention is made of any of this in the Abstract, or its significance given any relevance in the Discussion.

The results section has methodology that is repeated in the methods sections, and trying to look for specific information becomes difficult. Although this issue may be inherent to the nature of study, perhaps streamlining or combining methodology may be helpful.

The other aspect of the manuscript is the creation of the scFv and its impressive biological effects. Although there are omissions in this manuscript that detract from its thoroughness (see below), the vision of an scFv of an antibody as a heritable weapon against a tick-borne infection is far reaching and potentially consequential.

Abstract: Line 25. This study does not provide any information on “Having recently established novel protocols to genetically engineer the white-footed mouse, Peromyscus leucopus”. This sentence needs to be deleted.

Lines 150 – 169: The minimal (tdTomato) and the minimal-enhancer promoter (albumin) provided strong mRNA expression, but Supp Figure 1B shows that the minimal promoter led to greater expression. In the text, it is mentioned that the chosen construct was chosen to avoid overexpression. Something is not clear here. Wouldn’t it be better to overproduce antibody considering the weak response of the full-length construct?

Lines 200 – 207. The CMV promoter is very powerful but it is also known to be gradually silenced in long term cultures. Is this an issue that should be considered in a project where faithful heritability is of paramount importance?. Please identify the CMV promoter as the cytomegalovirus promoter. Line 315. Why wasn’t the CAG promoter used for expressing the full-length antibody? Use of this promoter may have yielded better results.

Lines 246 – 270. The parent LA-2 antibody is a potent agglutinator. Is it possible that the construct lost this ability leading to these poor results?

Figure 1B. Confusing figure. Try to improve to show heritability but poor and erratic antibody response. The notation for hetero-homozygous should be listed in the figure legends.

Lines 337 – 340. Methods for quantitation of antibody could not be found. Production of 0.25 and 0.50 mg/ml of scFv is very high, and could be associated with other biological effects. There is no size exclusion chromatography to determine the quaternary structure of the scFv. The 19 aminoacid (Line 302) linker that joins the VH and VL chains is designed for production of a monomeric scFv (AA Kortt et al 2001). However, scFvs intended to be monomers can also be capable of forming noncovalent multimers particularly in cases of high concentrations of scFvs, such as is seen here. While this scFv performed its intended biological tasks as expected, at least one of the unexplained results (elimination of spirochetes in ticks; Lines 407 – 412) could be explained by structural issues.

Throughout this manuscript, there are instances where there are no attempts to explain why certain choices for tests, strains or antibodies were made. Some of these are potentially important and could have affected the results.
1. The choice of monoclonal anti OspA LA-2 is never discussed. Although there is nothing wrong with the use of this antibody, there are others that are available and considering the variability of this lipoprotein, some clarification would be important. The provenance of the sequence of the antibody is not given. Was the original Wilske hybridoma (1988) used for PCR of the construct? Is the sequence published? Another feature that is not considered is that optimal protection was only achieved when LA-2 was given simultaneously but not after the spirochetal inoculation.

2. There is no mention of an irrelevant scFv control. Not absolutely essential but would have added rigor.

3. Lines 262 – 270. Why was the C6 serology assay used and not for example, whole cell ELISA or even the C6 supplemented by an immunoblot? Other assays could have provided better results for the mice expressing the full length knock-in.

Reviewer #3

(Remarks to the Author)
This is an interesting manuscript that reports heritable immunization technology directed towards the development of mice resistant to productive infection by B. burgdorferi, the Lyme disease spirochete. The punch line is that the authors have been successful in engineering a white-footed mouse stably expressing a single-chain antibody to OspA, which confers strong resistance to infection by B. burgdorferi in heterozygous mice providing the first step towards ecological engineering possibilities. In general, this is a well-written manuscript reporting high-quality science that pursues a creative idea. The conclusions are justified by the data and the manuscript describes a very significant advance. Some concerns regarding possible publication in Nature Communications are:
1) The first half of the manuscript focuses on the nuts and bolts of genetically engineering the desired mice. It represents a lot of essential work but is not interesting to read for the non-specialist.
2) The second half of the manuscript has the experimental results in two figures (Fig. 1 has negative data and is superfluous and more fitting for a Supplementary figure).
3) In lines 407-412 the authors note that the engineered antibodies do not kill spirochetes in the infected tick, in contrast to previous reports on OspA antibodies in immunized individuals (the basis of a previous Lyme vaccine by SmithKline Beecham and one currently in stage III clinical trials by Pfizer). This is an interesting result which could have been further investigated to add a bit more meat to the manuscript.

Minor issues:
1) After reading the Abstract I was left wondering what the target of LA-2 scFv-albumin fusion was. Some mention of OspA is needed in the Abstract.
2) Line 206 notes tat T2A was chosen from the elements tested. I was left wondering why, as it is not the best binder.

Reviewer #4

(Remarks to the Author)
The manuscript by Buchthal and colleagues describes the production of Borelleia neutralizing single chain antibodies in transgenic (Rosa26 Knockin) and their protection and blockage of pathogen transmission. In this immunization via the heritable trangene the population and environment incl. humans could be protected from the pathogen.
The study is easy to read well organized and the data and Figures support all claims. The study is the first proof of concept for heritable immunization which may be a very valuable strategy in future to control infction with mammalian pathogens in general. Therefor it will be of interest for a broader scientific community with focus on disease control and public health. However, I would recommend to lay down in the discussion the future possibilities to bring this into a population of wild mammals. It will be possible to directly manipulate a small fraction of the population; therefore it coul be combined with a CRISPR/Cas9 driven gene drive to spread the transgene in the population. Or can the authors envision some othe infective route to the germine e.g. by ERV like vectors able to infect male spermatogonial stem cells and integrate into the germline more randomly. Also which official regulations would need to be adapated for the release of the transgenic disease control ? Such discussion could lead the reader how to realize future scenarios of inherited disease control.

Reviewer #5

(Remarks to the Author)
I co-reviewed this manuscript with one of the reviewers who provided the listed reports. This is part of the Nature Communications initiative to facilitate training in peer review and to provide appropriate recognition for Early Career Researchers who co-review manuscripts.

Version 1:

Reviewer comments:

Reviewer #1

(Remarks to the Author)

The authors have responded carefully and thoughtfully to this reviewer's comments.

Reviewer #2

(Remarks to the Author)
NO FURTHER COMMENTSS FOR AUTHORS

Reviewer #3

(Remarks to the Author)
I am satisfied with the revisions made by the authors.

Reviewer #5

(Remarks to the Author)
I co-reviewed this manuscript with one of the reviewers who provided the listed reports. This is part of the Nature Communications initiative to facilitate training in peer review and to provide appropriate recognition for Early Career Researchers who co-review manuscripts.

Open Access This Peer Review File is licensed under a Creative Commons Attribution 4.0 International License, which permits use, sharing, adaptation, distribution and reproduction in any medium or format, as long as you give appropriate credit to the original author(s) and the source, provide a link to the Creative Commons license, and indicate if changes were made.
In cases where reviewers are anonymous, credit should be given to 'Anonymous Referee' and the source.
The images or other third party material in this Peer Review File are included in the article's Creative Commons license, unless indicated otherwise in a credit line to the material. If material is not included in the article's Creative Commons license and your intended use is not permitted by statutory regulation or exceeds the permitted use, you will need to obtain permission directly from the copyright holder.
To view a copy of this license, visit https://creativecommons.org/licenses/by/4.0/

Response to reviewers:

We thank the reviewers for their constructive feedback, which has undoubtedly improved our manuscript. We have carefully addressed each point raised, and where appropriate, have incorporated additional data and analyses. All revisions to the text are clearly highlighted in red.

Response to reviewer #1

Summary: More than 30 years have elapsed since the first reports that immunization with outer surface protein A (OspA) confers protection against Lyme disease. While the large majority of subsequent studies have focused on the development of OspA as a vaccine for humans, there also have been efforts to immunize reservoir hosts (typically white footed mice in North America) in the wild to interrupt the enzootic cycle that sustains Lyme disease spirochetes in nature. All of these studies have employed strategies to administer OspA to mice as a vaccinogen. This tautly written, clearly laid out study by Buchthal et al. takes a radically different and highly innovative approach: it uses state-of-the-art genetic engineering to create a murine mouse strain that heritably produces substantial levels of a single chain variable fragment derived from a well characterized murine monoclonal antibody (LA2) directed against a protective OspA epitope. The authors demonstrate convincingly that the strategy has great promise. Transmission of B. burgdorferi by infected nymphs is greatly diminished as is the ability of infected, heritably immunized mice to pass the bacterium on to feeding larvae. Nevertheless, some aspects of this impressive study relating mostly to the borreliology could be clarified and/or strengthened:

Major Comment 1: Lines 154 and Suppl. Fig. 1B. The text states that the constructs with and without the albumin enhancer produced comparable levels of tdTomato with and without the albumin enhancer. However, the figure appears to show much greater levels of tdTomato RNA from the construct containing the albumin enhancer.

We thank the reviewer for highlighting this ambiguity in the text, which could indeed be misinterpreted and may have contributed to confusion. Our intention was to indicate that in the first design lacking the additional albumin enhancer, tdTomato mRNA levels were comparable to those of endogenous albumin, rather than to suggest that the two designs produced equivalent levels of tdTomato expression. We meant to convey that albumin-level expression represents a high baseline, and that achieving similar expression without an added enhancer should be sufficient for robust protein production. We have revised the text to clarify this point more explicitly:

> Edits to the Results:
> RT-qPCR analysis revealed comparable levels of tdTomato and native albumin mRNA expression in the first cell line without the added enhancer (**Supp Fig. 1B**), suggesting that this more minimal design was capable of driving robust expression at levels comparable to endogenous albumin. Given the already high abundance of albumin in circulation, inclusion of the additional enhancer raised concerns about potential antibody overexpression. We therefore selected the first design for *in vivo* studies.

Major Comment 2: Lines 288-291 and Suppl Fig. 2. What were the sources of the antibodies used for these experiments?

We thank the reviewer for identifying this omission. We have added text to the Methods to clarify the source of the antibodies used for these experiments, along with additional experimental details.

> Edits to the Methods:
> **Bio-layer interferometry (BLI) assay**. Full-length LA-2 IgG2a plasmid was constructed by GenScript. To generate LA-2 scFv-Fc, heavy chain and light chain variable region genes of LA-2 were amplified from the

full-length plasmid and linked with a flexible (G4S)3 linker, the generated scFv was cloned into a modified pcDNA3.4 vector. LA-2 IgG2a and LA-2 scFv-Fc plasmid were separately transfected into Expi293F cells (Thermo Fisher Scientific, catalog #A14635) following the standard ExpiFectamine293 protocol. The supernatants were harvested and incubated with Protein A-Sepharose 4B resin (Invitrogen) for 3h at 4°C followed by purification via gravity flow columns (BioRad) and buffer exchanged by centrifugation in Amicon centrifugal filters.

Major Comment 3: Lines 358-383 and Figure 2D. The authors measure production of C6 antibodies as their indicator of transmission by feeding nymphs. However, this is a rather limited analysis. A more accurate picture of spirochete burdens could have been obtained by culture of ear punches and internal tissues as well as qPCR. The authors subsequently (lines 417-39) used xenodiagnosis to assess acquisition of spirochetes by feeding larvae. While acquisition was largely prevented, the results also showed that some mice were indeed infected, further arguing for a more robust characterization of burdens. Because OspA antibodies interfere with acquisition (as shown here), these results do not directly speak to the spirochete burdens in the mice upon which the larvae fed.

We thank the reviewer for this thoughtful suggestion. We agree that culture- or qPCR-based assessment of spirochete burdens in host tissues can provide additional information about infection status. However, the primary objective of this study was to assess reservoir competence rather than host susceptibility to *Borrelia burgdorferi*.

Accordingly, we focused on xenodiagnosis as the most relevant functional readout, since transmission to ticks is the critical parameter for evaluating strategies aimed at reducing pathogen circulation in nature. Seroconversion (C6 antibody production) was used as an indicator of host exposure following feeding by infected nymphs. We further note that qPCR-based assays detect Borrelia DNA rather than viable spirochetes and therefore do not directly measure host infectiousness, and that tissue spirochete burdens correlate imperfectly with reservoir competence.

Because the purpose of the antibody is to interfere with acquisition by feeding ticks, reduced transmission to ticks represents the most meaningful endpoint. Thus, the xenodiagnostic challenge was not intended to quantify spirochete burden within the mouse, but rather to measure the functional reduction in reservoir competence, which is the key outcome of interest.

We thank the reviewer for prompting this clarification. We have revised the text to explicitly frame xenodiagnosis as an assessment of transmission potential rather than mouse infection burden. We have also noted in the Discussion that direct measurements of tissue spirochete burdens were not performed; while such analyses may be informative for elucidating mechanism of action, they fall outside the scope of the present study.

Edits made to the Results:

Testing *Borrelia* Transmission to Uninfected Ticks Feeding on Previously Challenged Rosa26-Targeted LA-2 scFv-Albumin Mice

Even without *Borrelia* clearance from already-infected ticks, engineered mice may reduce or block transmission to the next generation of larvae. To assess reservoir competence and the impact of anti-OspA antibodies on *Borrelia* transmission, we performed xenodiagnosis using uninfected larval ticks (**Fig. 3A**)[27].

Edits made to the Discussion:

While our results demonstrate effective immune protection, the precise mechanism remains unclear and warrants further study. We note that tissue spirochete burdens were not assessed by culture or qPCR in this study, as our analyses focused on transmission outcomes rather than host infection burden.

Edits made to the Methods:

Xenodiagnosis. To assess the ability of mice to transmit *Borrelia burgdorferi* to feeding ticks, xenodiagnosis was performed[27].

Major Comment 4: Lines 388-412 and Figure 2E. An important aspect of the paper is the effect of the secreted antibody fragment on spirochetes in infected, feeding nymphs. The authors anticipated that the antibody would be borreliacidal although that is not what they believe they observed. However, the data provided is not easily interpretable since they relied entirely upon IFA rather than quantitative culture of spirochetes from nymphal midguts. Moreover, the presentation of the IFA data is confusing. The Methods (lines 1028-1032) states that they graded the IFAs on a scale of 0-4. The Results, on the other hand, states that “each tick was categorized based on the presence or absence of spirochetes regardless of infection intensity”, while Figure 2E shows, according to the labeling of the Y axis, “the fraction of infected ticks per challenged mouse". The wording here is obscure, but it does not indicate quantitation. The explanation of the results provided in the Discussion (lines 476-487) further adds to the confusion because it largely centers on older data relating to reciprocal expression of OspA and OspC rather than more recent studies of how spirochetes migrate through the midgut. Along these lines, it should be noted that the mechanism whereby OspA antibodies prevent transmission is far from clear; not all investigators find that OspA antibodies, including LA2, are borreliacidal.

We thank the reviewer for identifying inconsistencies in the description and presentation of these data. We agree that the description of how ticks were scored in the Results and Methods was not consistent, and we have revised the text throughout to address this.

For the analyses presented in Fig. 2E, the readout was intentionally binary: individual ticks were scored solely on the presence or absence of detectable *Borrelia burgdorferi*. Although immunofluorescence staining was initially evaluated qualitatively for spirochete morphology and relative density, these features were not used as outcome measures in this study. We have revised the text to consistently reflect that ticks were categorized as infected or uninfected, irrespective of infection intensity.

With respect to quantification, we acknowledge that culture of nymphal midguts can provide sensitive detection of low-level infection. However, culture outcomes depend strongly on incubation duration and can amplify very small inocula that may not persist within the tick during post-feeding development and molting. Because ingested antibody is likely to continue acting (however it acts, likely not just by destroying spirochetes) within the gut throughout digestion and molting, rare spirochetes that might be recoverable by culture at day 14 or day 21 post feeding may not survive within the native tick environment, either due to eventual destruction or loss/digestion due to failure to bind to gut receptors and successful gut colonization. Because our analyses focused on transmission outcomes in the native tick–host system rather than on quantitative outgrowth under artificial culture conditions, we elected not to perform culture-based assays.

We agree that the mechanism by which OspA-directed antibodies interfere with transmission remains incompletely understood. As suggested by the reviewer, we have incorporated discussion of more recent studies examining spirochete transmigration through the tick midgut as a potential stage of the transmission process that may be disrupted.

Together, these revisions clarify the binary nature of the readout used in Fig. 2E and elsewhere, and expand the Discussion to provide a more comprehensive consideration of potential modes of interference. We thank the reviewer for these constructive suggestions, which have helped improve the clarity and framing of the manuscript.

> Edits made to the figure legend:
> E) Graph showing the fraction of Borrelia-infected ticks per individual mouse, comparing ticks that fed on transgenic versus wild-type mice. Engorged ticks were collected, stored, and assessed for the presence or absence of Borrelia spirochetes using indirect immunofluorescence.
>
> Revised text after removal of a sentence that implied qualitative scoring:
> For analysis, each tick was categorized based on the presence or absence of spirochetes, regardless of infection intensity (**Fig. 2E**).

Edits made to the Methods:
All slides were examined using epifluorescence at X400, and individual ticks were scored solely for the presence or absence of spirochetes.

Edits made to the Discussion:
By binding to the surface of Borrelia, anti-OspA antibodies may prevent the bacteria from reaching the critical density required for the OspA/OspC gene switch, or alternatively, they may impair the midgut transmigration essential for transmission[33]. To this end, more recent work has emphasized that successful transmission requires regulated dissemination of spirochetes from the tick midgut during feeding, and that disruption of this process can substantially impair transmission[34,35].

Major Comment 5: The Discussion of next steps is candid and thoughtful. However, the statement "Our recent advances in rodent ovulation tracking…" (lines 521-524) needs clarification.

We thank the reviewer for noting that this statement required clarification. We have revised the text to specify that these advances refer to recently developed non-invasive ovulation tracking methods that enable genetic modification in non-model rodent species, and we have added a citation to our recent work describing this approach (currently available as a bioRxiv preprint and accepted for publication in Cell Reports Methods).

Edits in the Discussion:
Our recent advances in rodent ovulation tracking, which enable non-invasive identification of fertile windows and facilitate genetic modification in non-model rodents, may open new avenues for engineering other non-model species involved in *Borrelia* transmission, offering a potential strategy for controlling Lyme and other diseases involving rodent reservoirs[36].

Response to reviewer #2

Summary: The report ¨Heritable immunization of mice against Lyme disease enables ecological disease prevention¨ by a large group led by Joanna Buchthal describes the creation and the characterization of a single chain antibody (scFv) to Borrelia burgdorferi that was engineered into laboratory mice. Several generations of engineered mice produced scFv's capable of binding the parent antigen (OspA) and protecting mice against experimental infection via tick bites. This report clearly demonstrates the feasibility of creating heritable immunization – a preamble to multiple applications. This report also provides a step by step description of the creation of the full-length antibody expression in mice that did not produce appreciable immunity, and was thus abandoned. By any measure, this report is a robust feat of genetics with far-reaching possibilities. The authors should be commended for not overstating the field (disease control) applications of their work as these can present both foreseen and unforeseen challenges.

General Comment 1: The format of this manuscript leads to some issues of readability. One aspect deals with the negative (but important) aspects of engineering a full-length antibody. This manuscript first delves into the generation and validation of liver-specific full-length LA-2 antibody expressing mice. This part of the manuscript comprises the supplemental figure data as well as data in Figure 1. As mentioned, the mice engineered with full length antibody showed erratic production of the antibody as well as a response to challenge. Hence, the effort of creating mice expressing a full-length antibody provided a negative result. Yet, the methodology for its creation occupies a large amount (one half) of space in the manuscript and no mention is made of any of this in the Abstract, or its significance given any relevance in the Discussion.

We thank the reviewer for this thoughtful comment and agree that the section of the Results documenting the full-length antibody engineering efforts warrants mention elsewhere in the manuscript, given the substantial experimental effort involved and the important negative results that informed the final scFv–albumin design. We have revised the Abstract and Discussion to briefly acknowledge this work and clarify its role in guiding selection of the antibody format ultimately used.

> Revised Abstract:
> Engineered mice stably produced a LA-2 scFv-albumin fusion protein targeting the *Borrelia* outer surface protein A (OspA) across multiple generations, demonstrating robust heritability and stability of gene expression after optimization of antibody format.
>
> Edits to the Discussion:
> By encoding anti-Lyme antibody genes into the germline of *Mus musculus*, we conferred heritable resistance to Lyme disease. After evaluating multiple antibody formats, including full-length and reformatted constructs, the selected antibody design was stably inherited across multiple generations and provided robust immunity to infection.

General Comment 2: The results section has methodology that is repeated in the methods sections, and trying to look for specific information becomes difficult. Although this issue may be inherent to the nature of study, perhaps streamlining or combining methodology may be helpful.

We thank the reviewer for raising this important point regarding the clarity and organization of the Results and Methods sections. We carefully reviewed the manuscript with this concern in mind and streamlined the Results to reduce methodological detail that was more appropriately placed in the Methods. Specifically, we removed references to particular cell lines, detailed ELISA procedures, and aspects of the tick challenge protocol that could be relocated without compromising interpretation of the data, as well as a section describing antibiotic selection in cell lines. We also removed redundant text from the Methods where possible, although some limited repetition was retained to maintain clarity and context. In addition, the description of the initial infected tick challenge was relocated to better align with the flow of the Results, and experimental descriptions throughout the manuscript were simplified to improve accessibility for a general audience. Given the extent of these changes, we have not enumerated each edit here, but all revisions are reflected in the updated manuscript.

Additional Summary: The other aspect of the manuscript is the creation of the scFv and its impressive biological effects. Although there are omissions in this manuscript that detract from its thoroughness (see below), the vision of an scFv of an antibody as a heritable weapon against a tick-borne infection is far reaching and potentially consequential.

Major Comment 1: Abstract: Line 25. This study does not provide any information on “Having recently established novel protocols to genetically engineer the white-footed mouse, Peromyscus leucopus ”. This sentence needs to be deleted.

We thank the reviewer for this comment and agree that the referenced study was not clearly presented in the original text. We have addressed this by expanding the Discussion to describe these recent methodological advances. The highlighted protocols which enable genetic engineering of the white-footed mouse (Peromyscus leucopus) are described in our manuscript that is currently available as a bioRxiv preprint and has been accepted for publication in Cell Reports Methods.

> Edits to the Discussion:
> While our findings represent a critical first step toward heritable immunization, several challenges remain before implementation. Foremost among these is the need to engineer *Peromyscus leucopus* to carry heritable immunity. We have now established the ability to genetically engineer *Peromyscus*[36], but antibody constructs have not yet

been genomically encoded in this species to recapitulate the effects described here.

Additional edits to the Discussion:
Our recent advances in rodent ovulation tracking, which enable non-invasive identification of fertile windows and facilitate genetic modification in non-model rodents, may open new avenues for engineering other non-model species involved in *Borrelia* transmission, offering a potential strategy for controlling Lyme and other diseases involving rodent reservoirs[36].

Major Comment 2: Lines 150 – 169: The minimal (tdTomato) and the minimal-enhancer promoter (albumin) provided strong mRNA expression, but Supp Figure 1B shows that the minimal promoter led to greater expression. In the text, it is mentioned that the chosen construct was chosen to avoid overexpression. Something is not clear here. Wouldn't it be better to overproduce antibody considering the weak response of the full-length construct?

We thank the reviewer for this comment and appreciate the opportunity to clarify this point. We have revised the text to make clear that our concern regarding overexpression was relative to endogenous albumin levels, given that albumin is the most abundant serum protein. In the in vitro comparison, the minimal promoter alone, without the added albumin enhancer, drove expression at levels comparable to native albumin, indicating that this design was already sufficient for very high expression. Given this baseline, inclusion of the albumin enhancer raised concerns about excessive antibody production rather than insufficient expression, and we therefore selected the minimal design for in vivo studies.

Edits to the Results:
RT-qPCR analysis revealed comparable levels of tdTomato and native albumin mRNA expression in the first cell line without the added enhancer (**Supp Fig. 1B**), suggesting that this more minimal design was capable of driving robust expression at levels comparable to endogenous albumin. Given the already high abundance of albumin in circulation, inclusion of the additional enhancer raised concerns about potential antibody overexpression. We therefore selected the first design for *in vivo* studies.

Major Comment 3: Lines 200 – 207. The CMV promoter is very powerful but it is also known to be gradually silenced in long term cultures. Is this an issue that should be considered in a project where faithful heritability is of paramount importance? Please identify the CMV promoter as the cytomegalovirus promoter. Line 315. Why wasn't the CAG promoter used for expressing the full-length antibody? Use of this promoter may have yielded better results.

We thank the reviewer for raising these points and agree that clarification is needed. The use of the CMV promoter was limited to a short-term *in vitro* assay designed specifically to compare bicistronic elements (2A peptides and IRES sequences) for coordinated heavy- and light-chain expression. In this context, CMV was selected because it provides robust transient expression over the short time frame required for these comparisons and was not intended for long-term or heritable expression.

We have revised the text to explicitly state that this experiment was performed *in vitro* and that CMV was used solely for transient expression in mammalian cells, such that concerns about promoter silencing are not relevant in this context. To be clear, CMV was not used in any of the mouse models.

Revised text in the Results:
Bicistronic Element Selection
To achieve expression of the full-length LA-2 antibody, we utilized bicistronic elements commonly employed in antibody expression cassettes to produce both heavy and light chains without additional expression machinery. We evaluated several 2A peptides and internal ribosome entry sites (IRES) to determine which element best facilitated the coordinated expression of both chains *in vitro*. We built five expression constructs that each featured a cytomegalovirus (CMV) promoter driving expression of the LA-2 heavy chain adjacent to a distinct 2A

or an IRES sequence followed by the LA-2 light chain. Constructs were transiently transfected and ELISAs were performed on antibody supernatant to evaluate antibody concentration and binding.

With respect to the full-length antibody mouse model, we did not use the CAG promoter because our initial design goal was liver-specific secretion into the bloodstream, for which the albumin promoter was selected as the strongest available liver-specific promoter. Only later did it become clear that this strategy was inadequate for stable, high-level expression of a full-length antibody, motivating the use of the stronger ubiquitous CAG promoter in the subsequent mouse design. We have clarified this rationale in the revised text.

Revised text in the Results:
To develop a heritable immunization strategy against Lyme disease, we began by optimizing the expression of the anti-*Borrelia burgdorferi* antibody LA-2 in mouse hepatocytes. We aimed to minimize unintended physiological effects by restricting antibody expression to a specific tissue while leveraging the native albumin expression machinery to drive antibody secretion into the bloodstream.

Major Comment 4: Lines 246 – 270. The parent LA-2 antibody is a potent agglutinator. Is it possible that the construct lost this ability leading to these poor results?

We thank the reviewer for this thoughtful question. The full-length LA-2 antibody is a potent agglutinator, and reformatting the antibody could plausibly alter this property. While agglutination was not directly assayed in this study, the binding characteristics of the reformatted construct are consistent with a reduced capacity for multivalent interactions. Reformatting the full-length antibody into a single-chain variable fragment necessarily reduces binding valency from two antigen-binding sites to one, thereby limiting the capacity for antibody-mediated cross-linking. Following the reviewer's recommendation (see response to Major Comment 6), we performed size-exclusion chromatography, which indicated that the scFv–albumin construct behaves predominantly as a monomer under native conditions, without evidence of higher-order self-association that could otherwise increase effective binding valency and support agglutination. In addition, bio-layer interferometry revealed an approximately one-log reduction in binding affinity upon conversion of the full-length LA-2 to the scFv format (Supplementary Fig. 2), which may further contribute to diminished agglutinating potential. Together, these observations indicate that antibody format can influence functional properties beyond antigen binding alone and provide a plausible explanation for the absence of detectable borreliacidal activity in this context. The Discussion now notes that antibody reformatting may impact agglutination and offers a plausible explanation for the observed results.

Edits to the Discussion:
Without Fc-mediated effector functions, the LA-2 scFv appears sufficient to prevent *Borrelia* from establishing infection in the mammalian host, highlighting that antibody binding alone, independent of Fc-driven immune mechanisms, could effectively disrupt the transmission cycle. Differences in affinity, avidity, and binding valency between the scFv and the full-length antibody, including reduced agglutinating potential following reformatting, may together contribute to these distinct findings and help explain the absence of detectable borreliacidal activity despite preserved transmission-blocking efficacy. Future iterations incorporating new antibody formats designed to enhance affinity and avidity (as well as the creation of a non-*Borrelia* targeting scFv control mouse for comparison) could be explored for increased efficacy, particularly in applications requiring bactericidal activity or targeting other pathogens, and to further elucidate the underlying mechanisms of protection.

Major Comment 5: Figure 1B. Confusing figure. Try to improve to show heritability but poor and erratic antibody response. The notation for hetero-homozygous should be listed in the figure legends.

We appreciate the reviewer's feedback and made a series of changes to figure 1B to improve interpretability and visual accessibility for a broader audience. Specifically, we added descriptive titles above each genotype group to immediately

orient the reader to the dataset shown below, and clarified the x-axis to explicitly indicate that the bars represent serum concentrations for individual mice. We also repositioned the key to reduce visual emphasis and relocated the wild-type cohort, bringing the layout of Figure 1B into closer alignment with Figure 2B in order to improve consistency across antibody concentration plots.

In addition, to improve accessibility for a broader audience, we updated the genotype notation by explicitly pairing allelic shorthand with descriptive labels (e.g., “Heterozygous (+/KI)” and “Homozygous (KI/KI)”). This change has also been incorporated into the legend, and the same convention has been applied across all the other figures.

Updated Figure 1B in the Results:

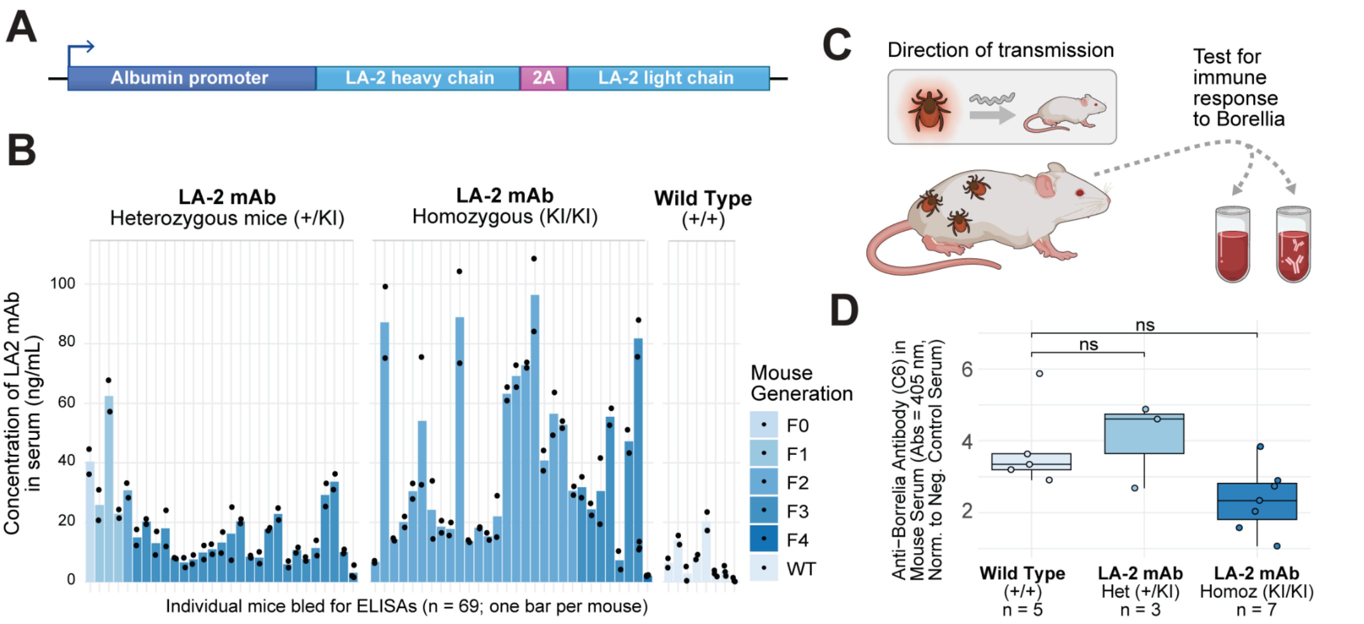

Figure 1. Generation, Validation, and Infection Challenge of Liver-Specific Full-Length LA-2-Expressing Mice
A) Schematic of the construct introduced into the mouse genome, 300 bp proximal to the native albumin enhancer. The minimal albumin promoter drives the expression of the full-length LA-2 monoclonal antibody. **B)** Quantification of LA-2 antibody concentration in the serum of heterozygous (+/KI), homozygous (KI/KI), and wild-type (+/+) mice across multiple generations (F0 through F4). **C)** Diagram of the tick challenge. Uninfected engineered and control mice were exposed to *Borrelia burgdorferi*-infected ticks, and post-infestation serum was evaluated for anti-*Borrelia* antibodies to determine infection status. **D)** Endpoint ELISA results from transgenic and wild-type mice 21 days post-infestation, detecting *B. burgdorferi*-specific antibodies against C6, a sensitive marker of infection.

Major Comment 6: Lines 337 – 340. Methods for quantitation of antibody could not be found. Production of 0.25 and 0.50 mg/ml of scFv is very high, and could be associated with other biological effects. There is no size exclusion chromatography to determine the quaternary structure of the scFv. The 19 amino acid (Line 302) linker that joins the VH and VL chains is designed for production of a monomeric scFv (AA Kortt et al 2001). However, scFvs intended to be monomers can also be capable of forming noncovalent multimers particularly in cases of high concentrations of scFvs, such as is seen here. While this scFv performed its intended biological tasks as expected, at least one of the unexplained results (elimination of spirochetes in ticks; Lines 407– 412) could be explained by structural issues.

We thank the reviewer for pointing out that antibody quantitation was not specified. While the protocol used to detect circulating antibody levels was described in the Methods, the approach used to determine antibody concentration was not

clearly described. We have now clarified that a purified LA-2 scFv–albumin protein was used as a standard to enable absolute quantification of serum antibody levels. The Methods section has been revised accordingly.

Edit to the Methods:
Testing mouse serum for LA-2 scFv-Albumin. Serum samples were collected from mice using BD Microtainer serum separation tubes. ELISA plates were coated with rOspA (produced by GenScript) at a concentration of 5 μg/mL. After applying the serum samples to the coated plates, LA-2 scFv-Albumin levels were detected using the Mouse Albumin ELISA Kit (Bethyl Laboratories, E99-134) with an anti-albumin secondary antibody. As a standard, purified LA-2 scFv-Albumin-His (produced by GenScript) was used for quantification of circulating antibody levels.

We thank the reviewer for suggesting the use of size-exclusion chromatography (SEC) to assess the quaternary structure of the scFv. This analysis is important and potentially informative, given the possibility of noncovalent multimerization even with linkers commonly used in monomeric scFv formats. SEC analysis of the purified LA-2 scFv–albumin construct revealed that the reformatted antibody elutes predominantly as a single species, with no substantial evidence of higher-order oligomers under the conditions tested. These data indicate that the construct does not undergo stable self-association detectable by SEC, making it unlikely that the observed biological effects are driven by scFv multimerization. Instead, the absence of multimerization and the reduced effective binding valency may contribute to the lack of detectable borreliacidal activity observed in this study. Please find the corresponding updates to the manuscript below.

Edit to the Results:
Given the high circulating antibody concentrations observed, size-exclusion chromatography (SEC) was performed on purified LA-2 scFv-albumin to evaluate quaternary structure. The antibody eluted predominantly as a monomer (Supp Fig. 4), suggesting that these concentrations are unlikely to drive higher-order oligomerization or aggregation.

Edit to the Methods:
Size-exclusion chromatography (SEC-HPLC). Purified LA-2 scFv–albumin was analyzed by size-exclusion chromatography using a TSKgel G3000SWxl column (Tosoh Bioscience) on an HPLC system (Agilent 1260). Samples (47 μL) were injected and eluted isocratically using 0.1 M Na_2SO_4 in 0.118 M phosphate buffer (pH 6.7 ± 0.3) as the mobile phase. Elution was monitored by UV absorbance at 280 nm, and chromatograms were analyzed using the HPLC system software. Molecular weight standards were run under identical conditions.

New Supplemental figure and legend:

A

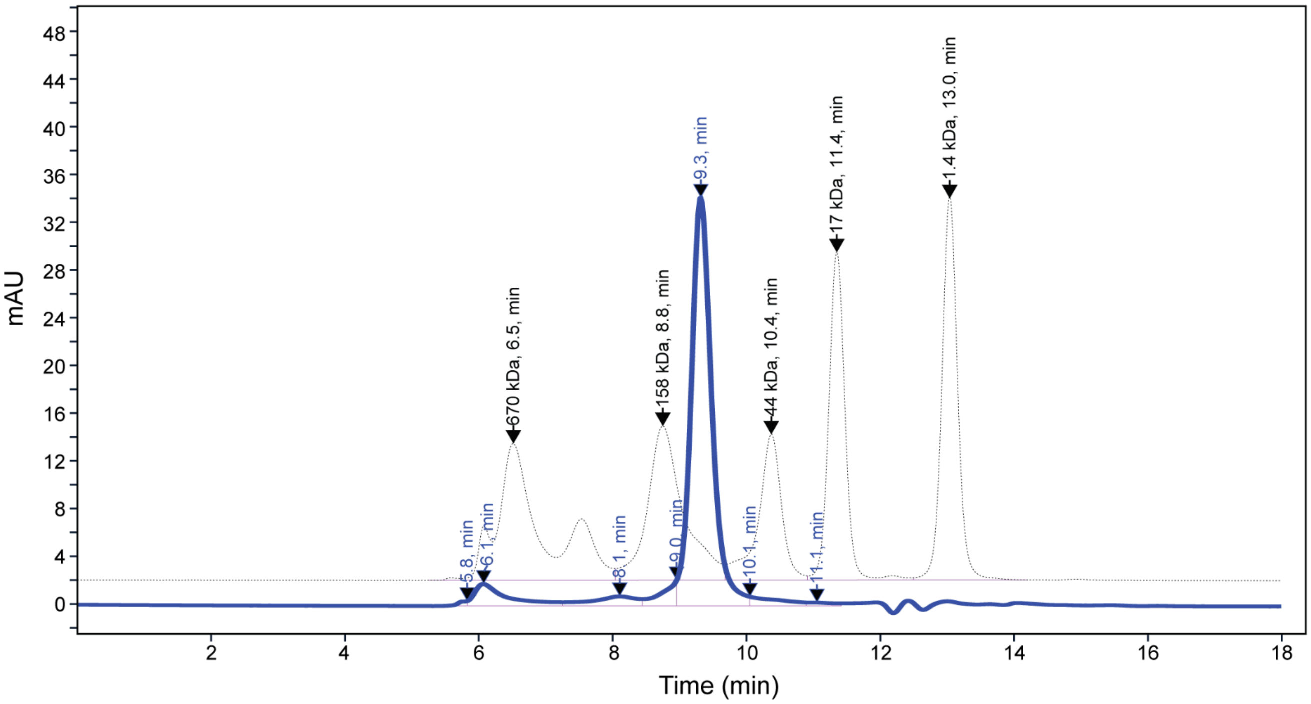

B

RT (min)	Height	Width (min)	Area	Area %
5.8	0.4	0.7	3.5	0
6.1	1.8	1.4	64.7	7
8.1	0.8	1.2	37.7	4
9.0	2.1	0.5	32.7	4
9.3	34.2	1.1	740.2	81
10.1	0.8	0.8	24.6	3
11.1	0.3	0.5	7.7	1
		Sum	911.12	

Supplemental figure 4. Size-exclusion chromatography of LA-2 scFv–albumin
A) Size-exclusion chromatography (SEC) profile of purified LA-2 scFv–albumin. **B)** Summary table corresponding to the chromatography profile shown in (A).

Throughout this manuscript, there are instances where there are no attempts to explain why certain choices for tests, strains or antibodies were made. Some of these are potentially important and could have affected the results.

Minor Comment 1: The choice of monoclonal anti OspA LA-2 is never discussed. Although there is nothing wrong with the use of this antibody, there are others that are available and considering the variability of this lipoprotein, some

clarification would be important. The provenance of the sequence of the antibody is not given. Was the original Wilske hybridoma (1988) used for PCR of the construct? Is the sequence published? Another feature that is not considered is that optimal protection was only achieved when LA-2 was given simultaneously but not after the spirochetal inoculation.

We thank the reviewer for highlighting these omissions and agree that the choice and provenance of the LA-2 monoclonal antibody should be explicitly addressed. We have revised the manuscript to clarify both the source of the antibody sequences and the rationale for selecting LA-2.

LA-2 was selected because it is among the most extensively characterized anti-OspA monoclonal antibodies, with a well-defined protective epitope and a published heavy- and light-chain variable region sequence. LA-2 served as a benchmark correlate of protection during early OspA vaccine development and was used in competitive inhibition assays to quantify protective anti-OspA responses in the LYMErix vaccine trials. Its extensive prior use therefore provides a well-established reference point for evaluating transmission-blocking immunity in vivo.

The LA-2 heavy- and light-chain variable region sequences used in this study were derived from the published structural characterization of the LA-2 monoclonal antibody reported by Ding et al. (2000), which analyzed an antibody generated from the original LA-2 hybridoma.

Moreover, prior work has shown that LA-2 confers optimal protection when present at the time of tick feeding rather than following spirochetal inoculation. This feature is directly relevant to the present study, as heritable immunization ensures continuous antibody presence during tick feeding, rather than relying on post-exposure intervention. We thank the reviewer for highlighting this important point.

We have revised the Introduction to contextualize the selection of LA-2 and added the source of the antibody genes to the Methods.

> Edits to the Introduction:
> In this study, transgenic *Mus musculus* were engineered to express LA-2, a *Mus*-derived monoclonal antibody targeting OspA[17], with the aim of disrupting Lyme disease transmission. LA-2 was chosen because it has been extensively characterized at both the structural[18] and functional levels[19], and confers protection when present during tick feeding, a feature well aligned with heritable immunization[8].
>
> Edits to the Methods:
> **Antibody sequence design.** LA-2 heavy- and light-chain variable region sequences were obtained from the published LA-2 antibody structure[18] and used to generate both full-length and scFv antibody formats.

Minor Comment 2: There is no mention of an irrelevant scFv control. Not absolutely essential but would have added rigor.

We thank the reviewer for this thoughtful suggestion and agree that inclusion of an irrelevant scFv control would have further strengthened the study by helping to distinguish antigen-specific effects from potential nonspecific consequences of high-level antibody expression. At the time these experiments were conducted, generating and maintaining an additional transgenic mouse line expressing an irrelevant scFv was not feasible due to the substantial time and resource requirements associated with producing, validating, and breeding a new heritable line across multiple generations. In addition, the tick challenge experiments are inherently capacity-limited and time-intensive, and inclusion of an additional control line would have required either substantially extending experimental timelines or reducing the number of wild-type controls.

Importantly, the protective effects observed here are consistent with the well-established antigen specificity of LA-2 and are recapitulated in the scFv-expressing mice. Nonetheless, we agree that inclusion of an irrelevant scFv control would be valuable in future studies and have noted this as a limitation of the current work.

> Edit to Discussion:
> Future iterations incorporating new antibody formats designed to enhance affinity and avidity, as well as inclusion of an irrelevant scFv control, could be explored for increased efficacy, particularly in applications requiring bactericidal activity or targeting other pathogens, and to further clarify the underlying mechanisms of protection.

Minor Comment 3: Lines 262 – 270. Why was the C6 serology assay used and not for example, whole cell ELISA or even the C6 supplemented by an immunoblot? Other assays could have provided better results for the mice expressing the full length knock-in.

We thank the reviewer for the suggestion that additional serologic assays could have provided complementary information. However, the goal of this particular test was narrowly defined: to determine whether mice had become infected with *Borrelia burgdorferi* following tick challenge. For this purpose, seroconversion to the C6 peptide, a validated peptide antigen derived from VlsE, provides a sensitive and specific readout of host infection, rather than antibody breadth or vaccine- or antibody-induced responses.

Although two-tiered serologic testing (STTT or MTTT) is required for clinical diagnosis in humans, this framework is not necessary for controlled experimental infections in mice. Importantly, a whole-cell sonicate ELISA would have been confounded in this context, as it would detect anti-OspA antibodies produced by the engineered mice themselves and therefore could not reliably distinguish exposure or infection status.

Seroconversion unambiguously implies infection. Mice were bled prior to challenge and again one month after exposure to infected ticks. By this time point, even delayed or low-level infections would be expected to elicit a detectable anti-C6 response. Thus, detection of anti-C6 antibodies in mice that were seronegative at baseline was interpreted as evidence of exposure. Because exposure does not necessarily equate to infectiousness, xenodiagnosis—rather than serology—served as the primary assay for assessing transmission competence.

We appreciate the reviewer's request for clarification and hope this explanation demonstrates that the selection of the C6 ELISA was a deliberate methodological decision, tailored to the specific goals of the study and the unique constraints of the engineered mouse models.

Response to reviewer #3

Summary: This is an interesting manuscript that reports heritable immunization technology directed towards the development of mice resistant to productive infection by B. burgdorferi, the Lyme disease spirochete. The punch line is that the authors have been successful in engineering a white-footed mouse stably expressing a single-chain antibody to OspA, which confers strong resistance to infection by B. burgdorferi in heterozygous mice providing the first step towards ecological engineering possibilities. In general, this is a well-written manuscript reporting high-quality science that pursues a creative idea. The conclusions are justified by the data and the manuscript describes a very significant advance. Some concerns regarding possible publication in Nature Communications are:

Major Comment 1: The first half of the manuscript focuses on the nuts and bolts of genetically engineering the desired mice. It represents a lot of essential work but is not interesting to read for the non-specialist.

We appreciate the reviewer's perspective and recognize that the early sections of the manuscript are technically detailed. These sections describe the first implementation of heritable, antibody-based disease protection in *Mus musculus*, which required addressing a number of technical and design considerations that have not been previously established. While the individual steps are necessarily technical, their inclusion is essential for defining the design principles of this new class of mouse models and for providing the context needed to interpret the biological results presented in the second half of the manuscript.

In response to this comment and to improve accessibility for non-specialist readers, we have streamlined the Results section by reducing repeated methodological detail and consolidating procedural descriptions into the Methods wherever possible. Specifically, we removed references to particular cell lines, detailed ELISA procedures, and aspects of the tick challenge protocol that could be relocated without compromising interpretation of the data, as well as a section describing antibiotic selection in cell lines. In addition, experimental descriptions throughout the manuscript were simplified to improve accessibility for a general audience. These changes improve readability while preserving the rigor required for reproducibility. Given the extent of these revisions, we have not enumerated each edit here, but all changes are reflected in the updated manuscript.

Major Comment 2: The second half of the manuscript has the experimental results in two figures (Fig. 1 has negative data and is superfluous and more fitting for a Supplementary figure).

We respectfully disagree that Fig. 1 would be more appropriate for the Supplementary Information. While the results do not demonstrate robust protection, they are not purely negative; rather, they illustrate intermediate or partial outcomes that define the feasibility and constraints of this approach. In addition, Fig. 1 introduces the experimental readouts used throughout the study to evaluate immunity, providing important context for subsequent analyses. Together, these data show that heritable antibody-based disease protection is not achieved by default, but can be incrementally approached, and they motivate the design choices that lead to the successful strategy presented in Fig. 2. For these reasons, we believe Fig. 1 is most appropriately retained in the main text. To clarify its role, we have added the following text to the Results.

> Edits to the Results:
> Although some homozygous mice exhibited very low or undetectable C6 antibody levels, suggesting potential immunity, neither the homozygous nor heterozygous mice showed a statistically significant reduction in anti-C6 antibody levels compared to wild-type controls (**Fig. 1D**). Together, these intermediate results revealed key design constraints, established the framework used to evaluate immunity, and guided the development of the architecture described below.

Major Comment 3: In lines 407-412 the authors note that the engineered antibodies do not kill spirochetes in the infected tick, in contrast to previous reports on OspA antibodies in immunized individuals (the basis of a previous Lyme vaccine by SmithKline Beecham and one currently in stage III clinical trials by Pfizer). This is an interesting result which could have been further investigated to add a bit more meat to the manuscript.

We completely agree with the reviewer that the observation that anti-OspA antibodies did not produce an overt phenotype of spirochete destruction within infected ticks is interesting. This finding has implications for current clinical applications of anti-OspA immunity and warrants further investigation. However, a detailed mechanistic analysis of how anti-OspA antibodies act within the tick or host is beyond the scope of this work and will require dedicated future studies.

Importantly, the goal of the present study was not to dissect the molecular mechanism of anti-OspA activity, but to test whether genetically encoded anti-OspA antibodies can reduce reservoir competence in vivo. Our results demonstrate that

functional protection can be achieved even in the absence of clear evidence for spirochete killing within the tick, indicating that complete elimination of spirochetes is not required to block transmission.

To clarify this point, we have expanded the Discussion to outline additional potential mechanisms and future directions, while explicitly noting that these questions were not addressed experimentally in the present study.

Edits to the Discussion:
While our results demonstrate effective immune protection, the precise mechanism remains unclear and warrants further study. We note that tissue spirochete burdens were not assessed by culture or qPCR in this study, as our analyses focused on transmission outcomes rather than host infection burden. Previous work suggested that anti-OspA antibodies directly neutralize *Borrelia* in the tick midgut via direct cellular lysis or complement activation[8]. However, the single-chain antibody we employed did not clear *Borrelia* within the tick, suggesting that an alternative mechanism may be responsible for disrupting the transmission cycle. One plausible explanation, as proposed by Wang et al. (2016), is that anti-OspA antibodies interfere with *Borrelia*'s gene switch from OspA to OspC, which is essential for spirochete infection of vertebrates[19]. This switch, regulated by environmental signals (temperature, pH, and spirochete density) within the tick during a blood meal, is crucial for the successful infection of a mammalian host[32,33]. By binding to the surface of Borrelia, anti-OspA antibodies may prevent the bacteria from reaching the critical density required for the OspA/OspC gene switch, or alternatively, they may impair the midgut transmigration essential for transmission[33]. To this end, more recent work has emphasized that successful transmission requires regulated dissemination of spirochetes from the tick midgut during feeding, and that disruption of this process can substantially impair transmission[34,35].

Without Fc-mediated effector functions, the LA-2 scFv appears sufficient to prevent *Borrelia* from establishing infection in the mammalian host, highlighting that antibody binding alone, independent of Fc-driven immune mechanisms, could effectively disrupt the transmission cycle. Differences in affinity, avidity, and binding valency between the scFv and the full-length antibody, including reduced agglutinating potential following reformatting, may together contribute to these distinct findings and help explain the absence of detectable borreliacidal activity despite preserved transmission-blocking efficacy. Future iterations incorporating new antibody formats designed to enhance affinity and avidity (as well as the creation of a non-*Borrelia* targeting scFv control mouse for comparison) could be explored for increased efficacy, particularly in applications requiring bactericidal activity or targeting other pathogens, and to further elucidate the underlying mechanisms of protection.

Minor issues:
Minor Comment 1: After reading the Abstract I was left wondering what the target of LA-2 scFv-albumin fusion was. Some mention of OspA is needed in the Abstract.

We thank the reviewer for this suggestion. We have revised the Abstract to explicitly identify the target of the LA-2 scFv–albumin fusion by noting that it is directed against Borrelia burgdorferi outer surface protein A (OspA), making the antibody target clear at first mention. We appreciate the reviewer bringing this omission to our attention.

Edits to the Abstract:
After optimization of the antibody format, engineered mice stably produced a LA-2 scFv-albumin fusion protein targeting the *Borrelia* outer surface protein A (OspA) across multiple generations, demonstrating robust heritability and stability of gene expression.

Minor Comment 2: Line 206 notes that T2A was chosen from the elements tested. I was left wondering why, as it is not the best binder.

We thank the reviewer for this comment and agree that the question is reasonable without additional context. Indeed, T2A did not yield the highest binding among the 2A peptides tested. However, our selection of T2A was based on its overall performance and robustness rather than maximal binding alone. T2A is among the most widely used and well-characterized 2A peptides for bicistronic antibody expression and is known for robust and consistent cleavage across systems. In our hands, it enabled efficient co-expression and processing while maintaining sufficient antibody activity for functional protection. We have clarified this rationale in the text.

> Edits to the Results:
> T2A was chosen from the elements tested based on its overall performance and robustness rather than maximal binding alone (**Supp Fig. 1F**).

Response to reviewer #4

Summary: The manuscript by Buchthal and colleagues describes the production of Borelleia neutralizing single chain antibodies in transgenic (Rosa26 Knockin) and their protection and blockage of pathogen transmission. In this immunization via the heritable transgene the population and environment incl. humans could be protected from the pathogen.
The study is easy to read well organized and the data and Figures support all claims. The study is the first proof of concept for heritable immunization which may be a very valuable strategy in future to control infection with mammalian pathogens in general. Therefore it will be of interest for a broader scientific community with focus on disease control and public health.

Major Comment 1: However, I would recommend to lay down in the discussion the future possibilities to bring this into a population of wild mammals. It will be possible to directly manipulate a small fraction of the population; therefore it could be combined with a CRISPR/Cas9 driven gene drive to spread the transgene in the population. Or can the authors envision some other infective route to the germline e.g. by ERV like vectors able to infect male spermatogonial stem cells and integrate into the germline more randomly.

We thank the reviewer for the opportunity to discuss potential next steps toward population-level implementation, which we agree are both interesting and likely to be of broad interest. In contrast to applications that require rapid spread across large, well-mixed populations, Peromyscus leucopus exhibits limited dispersal, with most individuals remaining near their natal range and longer-distance movement largely restricted to a subset of young males, typically on the order of a few hundred meters. As a result, local populations are expected to remain spatially stable over extended periods.

In this ecological context, we do not anticipate that specialized genetic spread mechanisms, such as gene drives or infective germline vectors, will be needed. Instead, we envision small-scale, targeted releases in high-risk areas, such as residential environments, timed to seasonal lows in population size when natural bottlenecks occur. Under these conditions, introduced animals are expected to contribute disproportionately to subsequent population growth, allowing protective alleles to become prevalent locally through standard population dynamics.

Importantly, because heterozygous animals are protected, a single protective allele is sufficient to confer immunity, suggesting that protective effects could be maintained even in the absence of fixation. Together, these features support the feasibility of durable, localized impact through conventional release strategies informed by species ecology, rather than reliance on autonomous genetic spread technologies.

> Edits made to the Discussion:
> A release strategy will need to be established as this approach advances beyond laboratory settings. Given the limited dispersal of Peromyscus leucopus[38], population replacement through targeted releases, particularly during

seasonal bottlenecks, may be sufficient to achieve high frequencies of protective alleles without the need for gene drive-based approaches. Importantly, protection in heterozygous animals indicates that a single protective allele is sufficient to confer immunity, suggesting that protective effects could be maintained even in the absence of fixation (complete replacement of alternative alleles in the population).

Major Comment 2: Also which official regulations would need to be adapted for the release of the transgenic disease control? Such discussion could lead the reader how to realize future scenarios of inherited disease control.

We appreciate the reviewer's encouragement to address this topic, which we agree is of broad interest given that environmental release of genetically modified wild mammals is largely without precedent. While genetically modified insects have been approved for environmental release under existing regulatory frameworks, no comparable precedent exists for genetically modified wild mammals, which raise distinct ecological, ethical, and regulatory considerations related to longevity, mobility, reproductive biology, and societal expectations for vertebrate species. Although any such effort would clearly require coordinated regulatory oversight at the federal, state, and local levels, the precise regulatory pathways remain to be defined, even with input from advisors who have served in senior regulatory roles for genetically modified organisms in the federal government. Because these considerations extend beyond the scope of the present study, we anticipate addressing regulatory frameworks for heritable disease control in greater depth in future work, while briefly acknowledging these questions in the Discussion.

Edits made to the Discussion:
Because the intentional release of genetically modified mammals into the environment has no clear regulatory precedent, this application will likely necessitate new regulatory frameworks and adaptive approaches that integrate ecological risk assessment, public health goals, and community governance as this work moves toward real-world implementation.
